# Molecular Dynamics Force Field Parameters for the EGFP Chromophore and Some of Its Analogues

**DOI:** 10.1021/acs.jpcb.3c01486

**Published:** 2023-06-26

**Authors:** Kimberly L. Breyfogle, Dalton L. Blood, Andreana M. Rosnik, Brent P. Krueger

**Affiliations:** Hope College Department of Chemistry, Holland, Michigan 49423, United States

## Abstract

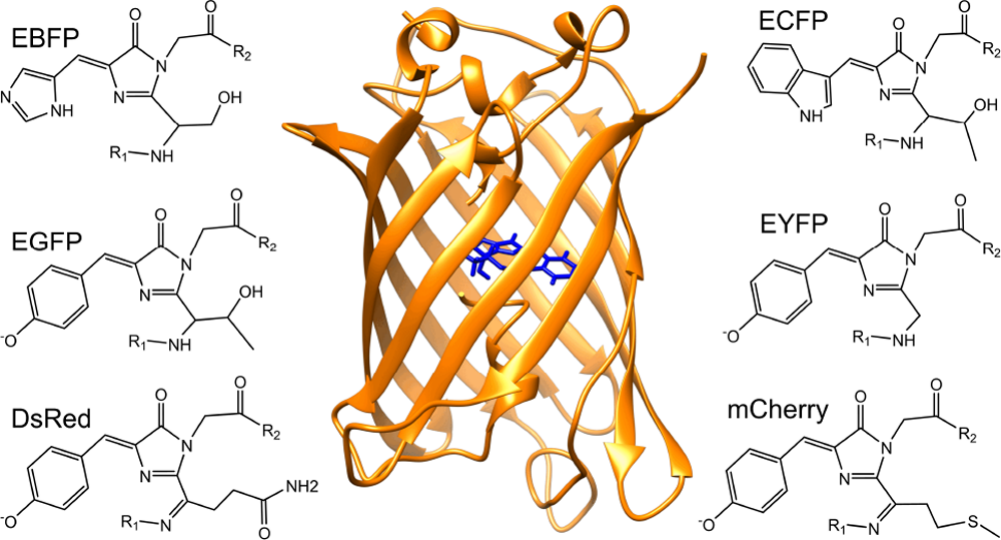

Fluorescent proteins (FPs) have had an enormous impact on molecular and cellular biology and are employed in a wide range of studies of molecular structure and dynamics. Yet, only a modest number of papers have published molecular dynamics (MD) parameters describing FPs. And despite the development of a wide range of FPs, there has been no careful development of MD parameters across a series of FPs. In this work, we present MD parameters describing six fluorescent protein chromophores (EGFP, EBFP, EYFP, ECFP, mCherry, and DsRed) for use with the Cornell *et al*. (J. Am. Chem. Soc.1995, 117, 5179−5197) family of AMBER force fields, including ff14SB and ff19SB. We explore a wide range of solvent dielectric constants for determining the chromophore equilibrium geometry and evaluate the impact of the modeled solvent on the final atomic charges. We also present our methodological approach in which we considered all six chromophores together with a focus on modularity, transferability, and balance with existing force fields. The parameters given here make it easy to employ MD simulations to study any of the six systems, whereas the methodology makes it easy for anyone to extend this work to develop consistent parameters for additional fluorescent proteins. The results of our own MD simulations are presented, showing that the classical MD parameters yield chromophore structural distributions that compare well with QM/MM simulations.

## Introduction

Green fluorescent protein (GFP) has revolutionized many scientific fields.^1−4^ Its utility has spurred the creation of a broad spectrum of related molecules with properties tuned for a variety of uses^5−17^ such that 25,900 papers were published in 2022 containing the phrase *fluorescent protein*.^18^ An increasing number of studies examine structural dynamics of the fluorescent protein (FP) chromophore and its surroundings such that molecular dynamics (MD) simulations are often employed (7440 of those 25,900 also contain the words *molecular*, *dynamics*, and *simulation*(18)). Thus, there is a need for MD parameters describing a spectrum of FPs. Below, we present a general methodology for chromophore parameter development that can be applied to any FP with a core structure similar to GFP or DsRed, we examine the dependence of the final atomic charges on the dielectric of the solvent used to determine the equilibrium geometry, and we apply our methodology to six FPs with emission wavelengths that range from blue to red, giving the resulting parameters.

One of the key reasons that FPs are powerful tools is the fact that the chromophore is generated from natural amino acids, making it easy to incorporate with nearly any protein using standard genetic tools. The three amino acids that are central to the chromophore undergo several chemical reactions that cyclize the backbone and extend π-conjugation, yielding a chromophore with absorption and emission (often strong) in the visible region of the spectrum.^4,5,10,11,19^ These chemical changes mean that the chromophores cannot be described by standard protein force fields, and new MD parameters must be developed. The McCammon group was the first to do this,^20^ describing the behavior of wild-type GFP with the Cornell *et al.*(21) force field used in AMBER. In the intervening years, a variety of groups have developed their own parameters or used those developed by others to describe GFP and several other FP varieties for use with several different force fields from the AMBER^22−30^ and CHARMM^31−36^ families. The MD parameters employed in each of those works was developed in a one-off fashion for that particular project and sometimes with charge models that do not match the underlying force field parameters. To our knowledge, no one has attempted to develop parameters meant to describe multiple FPs in a consistent way, a prerequisite to a parameter set that is easily extended to a wide range of FPs. Given the wide array of FPs that have been created and might be used in experimental studies, it would be valuable if MD parameters existed for a variety of FPs that are easy to utilize and internally consistent. Even more valuable would be a clear and consistent methodology that allows parameters to be easily developed for additional FP chromophores.

**Figure 1:**
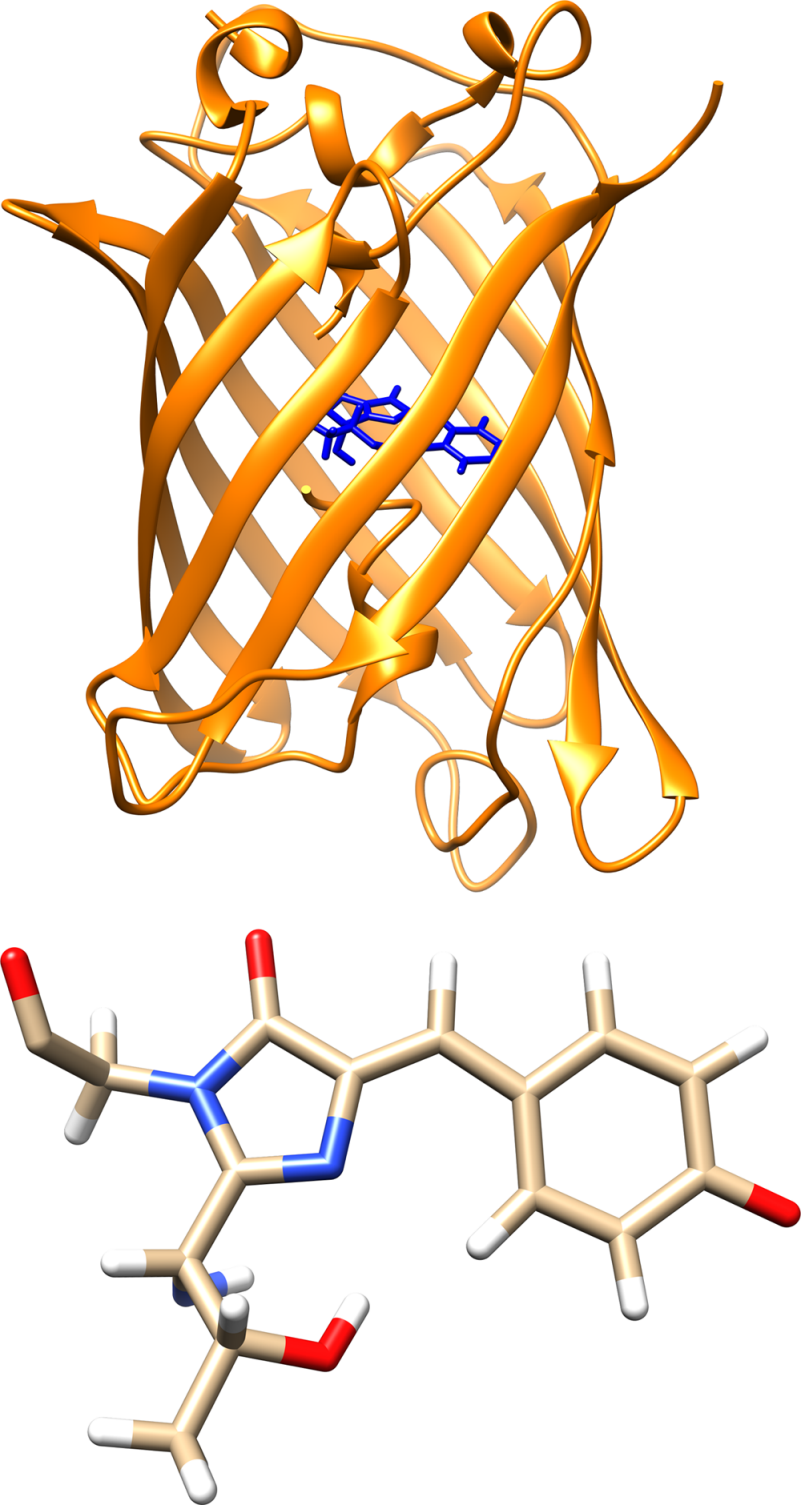
Upper: the EGFP protein. The chromophore (blue) of EGFP sits inside a water-excluding barrel of beta sheets (orange). Lower: a structural representation of the EGFP chromophore. Atoms are colored by element with tan carbon atoms, red oxygen atoms, blue nitrogen atoms, and white hydrogen atoms. Figure produced using Chimera.^43^

FPs like EGFP consist of a chromophore within a beta-barrel structure; see Figure 1. An entire field of discovery employs mutations in the chromophore and to nearby regions of the surrounding protein to control the emission properties of the resulting proteins. Properties such as the emission wavelength, pH dependence, and photostability can all be controlled and optimized.^6,7,11,14−17,37−42^

**Figure 2:**
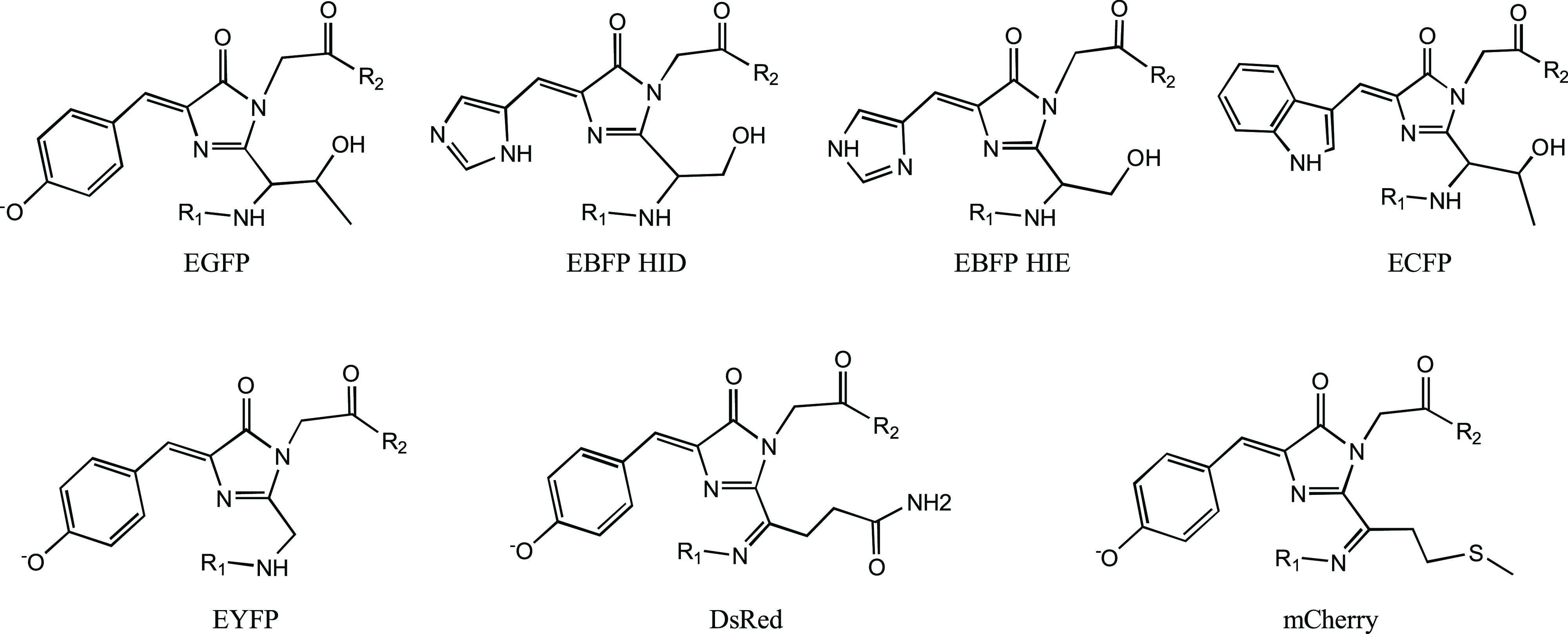
Chemical structures of the fluorescent protein chromophores presented in this work. Note that there are two forms of EBFP corresponding to the two neutral isomers of histidine.

In this work, we report MD parameters for six different fluorescent protein chromophores (EGFP, EBFP, EYFP, ECFP, mCherry, and DsRed with two forms of EBFP; see Figure 2) for use with ff14SB^44^ and ff19SB,^45^ the two newest versions of the family of Cornell-based^21^ AMBER force fields.^46^ All necessary parameters are included here as well as in the AmberTools package^47,48^ and so are freely available and can be easily implemented in any AMBER MD simulation. We also present our parameter development procedure that was applied across these chromophores with a focus on transferability and on maintaining balance with the ff14SB^44^ and ff19SB^45^ force fields. We treated the chromophores in a modular way such that there is a chromophore core onto which different sidechains are attached, keeping the parameters internally consistent and making it easy for anyone to extend this work to additional fluorescent protein chromophores in the future. Through this process, we examined several sets of force field parameters, comparing with quantum mechanical (QM) calculations to determine which best represented chromophore behavior. We also examined several QM solvent models, including dielectric constants ranging from vacuum to water to see their impact on chromophore structure and final partial atomic charges. Although the manuscript below will focus on EGFP, describing DsRed when needed, all six chromophores were considered equally in the parameter development process.

## Methods

### Chromophore Geometries

Reference structures were taken from the Protein Data Bank (PDB)^49^ for each of the six fluorescent proteins studied. Literature references were used to determine the protonation state that is believed to be present under physiological conditions. Like EGFP,^9,50^ the fluorescence for most of these chromophores (DsRed,^19,51^ EYFP,^52^ and mCherry^37^) is due to their anionic states, whereas EBFP^7^ and ECFP^12^ are thought to be neutral under physiological conditions. Table 1 provides a summary of this information for each fluorescent protein.

**Table 1:** Reference Information for the Six Fluorescent Proteins^a^

fluorescent protein	amino acids involved in chromophore	3-letter PDB code for chromophore	PDB reference structure	chromophore charge	chromophore protonation state references
EGFP	Thr-Tyr-Gly TYG	CRO	2Y0G	–1	(9,50)
EBFP	Ser-His-Gly SHG	IIC	1BFP	0	(7)
ECFP	Thr-Trp-Gly TWG	CRF	2WSN	0	(12)
EYFP	Gly-Tyr-Gly GYG	CR2	3V3D	–1	(52)
DsRed	Gln-Tyr-Gly QYG	CRQ	2VAD	–1	(19,51)
mCherry	Met-Tyr-Gly MYG	CH6	2H5Q	–1	(37)

a For each fluorescent protein, the amino acids that comprise the chromophore, abbreviation used for the chromophore in PDB Files, the PDB ID for the reference protein structure, the charge of the chromophore, and the reference(s) used to determine the appropriate protonation state are given.

For each protein, the structure of the chromophore plus one residue before and after was imported into the molecular editing program, WebMO.^53^ The leading and trailing residues were converted into acetyl (ACE) and *N*-methyl amide (NME) capping groups and the structures were then optimized using a four-step process. Each step utilized Gaussian16^54^ for the calculations with a solvent of 1-pentanol modeled with SMD^55^ (see the following paragraph). First, hydrogens were added manually and optimized using PM3;^56−60^ then hydrogens and capping group heavy atoms were optimized using the B3LYP level of theory^61−64^ with the 6-31G(d) basis set;^65,66^ all atoms were optimized using B3LYP/6-31G(d); and finally, all atoms were optimized once more using the same B3LYP/6-31G(d) model chemistry with “TIGHT” convergence criteria and an ultrafine DFT integration grid. All quantum mechanically optimized structures were overlaid with their crystal structures using the “match” function in *Chimera*(43) to align structures using the heavy atoms of the two rings, attached carbonyl oxygens, and the bridging atom (Figure 2). Figures showing these overlaid structures are available for each chromophore in the Supporting Information. Optimization had little effect on the two conjugated rings of the chromophores; atoms farther from the rings showed some displacement relative to the PDB structure, although they retained consistent configuration. The largest observed structural change was modest rotation of the first sidechain, seen in three chromophores (mCherry, DsRed, and ECFP). These sidechains remained consistent in shape but rotated slightly away from the nearby capping groups. All of the observed structural changes were modest, and those optimized structures were used for characterization of the electronic structure of the chromophores.

A wide range of solvent dielectrics (from 1.1 to 30) have been used to model protein environments in various studies over the past three decades,^67−80^ although most of these works have utilized ε = 10–20. Because of this ambiguity, we tested the full optimization procedure described above using six different solvents, including vacuum (ε = 1), pentylamine (ε = 4.20), 1-octanol (ε = 9.86), 1-pentanol (ε = 15.13), 2-propanol (ε = 19.26), and water (ε = 78.36), all implemented with the SMD solvent model.^55^ These solvents were chosen based on a number of factors: (a) in the crystal structure, there are a variety of sidechains within 3 Å of, and pointing toward, the chromophore including hydrophobic valine, aromatic/polar histidine, and multiple polar and/or charged groups including two threonines, arginine, glutamine, glutamic acid, and three waters, such that short-chain alcohols or amines are a reasonable compromise for mimicking this environment; (b) surface tensions in the range of 30–37 cal/mol·Å^2^, consistent with alcohols and amines of modest chain length; and c) dielectric constants that span the range of 4–20 plus the two extreme values of vacuum and water. Test optimizations were conducted on EGFP and mCherry, and we examined both the final structures as well as atomic charges calculated from those structures (using the RESP method, see below). The first three columns of Table 2 give root mean square (RMS) deviations of optimized structures for EGFP in all solvents. The vacuum structure is quite different from the others (first column, all RMS > 0.21 Å), whereas the other solvent structures are all within 0.16 Å of 1-pentanol (second column) and within 0.18 Å of water (third column). Similarly, the first three columns of Table 3 give RMS deviations of optimized structures of mCherry. In this case, both the vacuum- and water-optimized structures were significantly different from the others (all RMS > 0.22 Å), whereas the four solvents of moderate dielectric yielded essentially identical structures, all within 0.06 Å of the 1-pentanol structure. The last three columns of Tables 2 and 3 give the mean unsigned errors (MUEs) of the atomic charges that resulted from each of the structures. These final charges were very similar (MUE < 0.017e) for all solvents except vacuum. As can be seen in the second and fifth columns of Tables 2 and 3, the chromophore structures and atomic charges that resulted from optimizing with 1-octanol, 1-pentanol, and 1-propanol were all equivalent in both test chromophores. For this work, we have chosen 1-pentanol as our standard.

**Table 2:** Comparison of Structures and RESP Charges for the EGFP Chromophore Optimized in Solvents Ranging from Vacuum to Water^a^

SMD solvent model	RMSD of structure relative to vacuum (Å)	RMSD of structure relative to 1-pentanol (Å)	RMSD of structure relative to water (Å)	MUE of charges relative to vacuum (e)	MUE of charges relative to 1-pentanol (e)	MUE of charges relative to water (e)
vacuum	0	0.352	0.368	0	0.035	0.036
pentylamine	0.216	0.153	0.179	0.025	0.013	0.016
1-octanol	0.341	0.013	0.063	0.033	0.002	0.008
1-pentanol	0.352	0	0.057	0.035	0	0.007
2-propanol	0.352	0.091	0.112	0.032	0.006	0.007
water	0.368	0.057	0	0.036	0.007	0

a RMS deviations of structures and MUE of RESP charges are given using vacuum, 1-pentanol, and water-optimized structures as references.

**Table 3:** Comparison of Structures and RESP Charges for the mCherry Chromophore Optimized in Solvents Ranging from Vacuum to Water^a^

SMD solvent model	RMSD of structure relative to vacuum (Å)	RMSD of structure relative to 1-pentanol (Å)	RMSD of structure relative to water (Å)	MUE of charges relative to vacuum (e)	MUE of charges relative to 1-pentanol (e)	MUE of charges relative to water (e)
vacuum	0	0.259	0.592	0	0.046	0.048
pentylamine	0.225	0.057	0.439	0.044	0.007	0.017
1-octanol	0.248	0.016	0.433	0.046	0.001	0.015
1-pentanol	0.259	0	0.421	0.046	0	0.014
2-propanol	0.275	0.024	0.402	0.046	0.001	0.014
water	0.592	0.421	0	0.048	0.014	0

a RMS deviations of structures and MUE of RESP charges are given using vacuum, 1-pentanol, and water-optimized structures as references.

Additional geometry optimizations were performed on EGFP and DsRed to provide further data for atom type determination (see below) and to ensure that results were robust with respect to the details of the QM optimization. These further optimizations were conducted using MP2/cc-pVDZ model chemistry in solvents of vacuum, PCM(1-pentanol), and PCM(water) and also using B3LYP/6-31G(d) in vacuum, SMD(1-pentanol), and SMD(water). The resulting geometries differed only slightly from the original B3LYP/6-31G(d)/SMD(1-pentanol) geometries (no bond length differed by more than 0.025 Å), confirming that the level of theory and choice of surrounding dielectric do not have a large impact on chromophore structure.

### Atom Types and Force Field Parameters

Our primary goals in this work are as follows: (1) we want to develop MD parameters for the FP chromophores that are consistent with the family of force fields derived from the 1994 Cornell force field,^21^ particularly ff99SB,^81^ ff14SB,^44^ and ff19SB,^45^ which differ in only a modest number of parameters (and the addition of CMAPs for backbone dihedrals in ff19SB) and (2) we want these parameters to be as consistent as possible within the family of fluorescent proteins considered here, maximizing transferability and making it easy for us or others to develop parameters for other FP chromophores in the future. To maintain consistency and balance with the Cornell-based force fields, we sought to define the fewest possible number of new parameters. For those new parameters that would be needed, we chose to base them on either ff14/19SB or the generalized AMBER force field (gaff),^82^ which is designed to be compatible with the Cornell family of force fields. Although we have not developed CMAPs to describe the backbone dihedrals for these chromophores, ff19SB is designed to be compatible with non-CMAP residues by simply using the CX alpha-carbon atom type rather than the newer XC atom type.^83^ Thus, our final parameters are equally compatible with all of the Cornell-derived force fields that have been developed to date.

**Figure 3:**
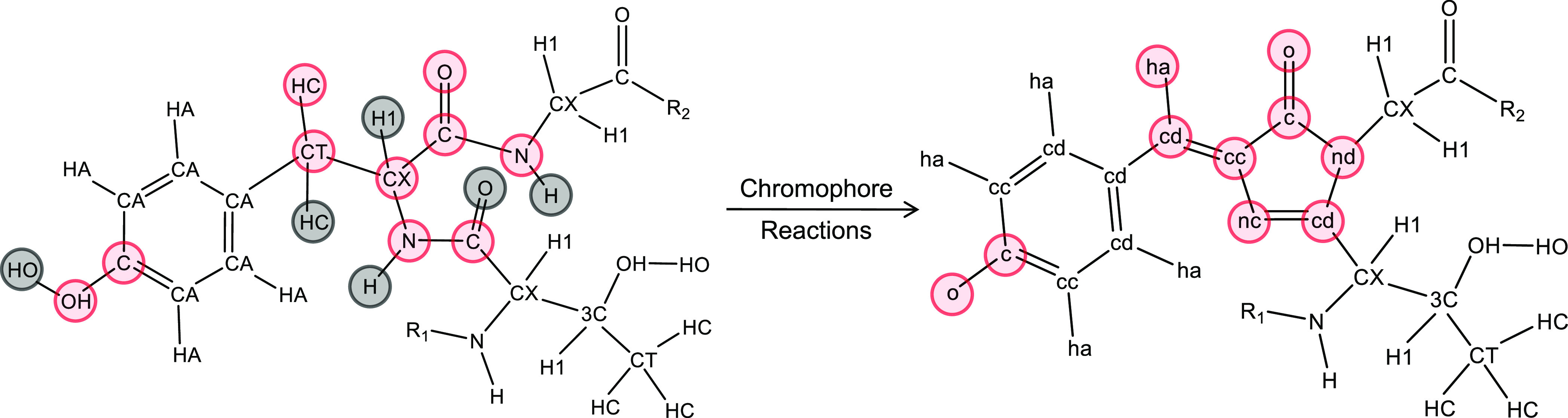
Left: structure and ff14SB atom types for the three amino acids that make up the EGFP chromophore. Right: structure and atom types from this work for the EGFP chromophore. Atoms shaded with a gray circle are removed during formation of the chromophore. Atoms shaded with a red circle are present in both structures but have had an obvious change to their chemical environment.

As a starting point, atom types for each chromophore were assigned based on the descriptions of the relevant amino acids in the ff14SB force field (which uses the same atom types as ff19SB aside from the CX/XC mentioned above). During the chemical reactions that form the chromophores, there are obvious changes to the bonding/chemical environments of many of the atoms as indicated in Figure 3. For atoms that have different bonding/environments in the chromophore relative to the parent amino acid, all reasonable atom types (based on bonding and chemical environment) were considered from both the generalized AMBER force field (gaff)^82^ and ff14SB. In most cases there was no clear reason to choose one parameter set over another based simply on description. Therefore, bond length parameters for the atom types under consideration were compared to quantum mechanically optimized bond lengths at multiple levels of theory and surrounding dielectric (see above) as well as to crystal structure bond lengths as described in Results and Discussion. Final atom types were assigned as described in Results and Discussion and shown in Figures 3 and 5 for EGFP and in Figure 5 for DsRed. Atom types for other chromophores are given in the Supporting Information.

Once atom types were determined, most bond, angle, dihedral, and van der Waals parameters were already defined by ff14SB or gaff and were used without adjustment. Any remaining (undefined) angle and dihedral parameters were assigned by analogy to ff14SB or gaff as described below in Results and Discussion. Parameters from gaff version 1.81 (May 2017) were utilized here. Note that, for the parameters utilized here, gaff 1.81 and gaff version 2 are identical with regard to all dihedral parameters and equilibrium angles (except c3-cf-ne, which differs by 1.03°), and their equilibrium bond lengths differ by less than 0.0005 Å. Interestingly, the bond force constants are all weaker in v2 compared to v1.81, whereas most of the angle force constants are stronger (one is unchanged). It is not clear to us that v2 parameters are likely to yield better results or that they would even be significantly different. Nonetheless, we will consider gaff 2 parameters in future work.

### Partial Charges

Atomic charges were determined using two-stage Restrained Electrostatic Potential (RESP) fits of electrostatic potentials (ESPs) that were computed on B3LYP/6-31G(d)/SMD(1-pentanol) optimized structures using HF/6-31G(d), as prescribed by Kollman and co-workers.^21,84−86^ This method takes advantage of the fact that vacuum Hartree–Fock results overpolarize the molecule to a degree that mimics the polarization one would expect to observe in protein solvated by water. We note that the highly conjugated chromophores studied here are expected to show more polarization due to solvation than standard amino acids and, therefore, they may not be approximated as well by charges determined using vacuum HF ESPs. The Grubmüller group has developed a procedure specifically for fluorescent molecules in which RESP charges are fit from an ensemble of ESPs determined using QM/MM simulations of the fluorophores in explicit solvent.^87^ These parameters were then implemented in AMBER by Schepers and Gohlke.^88,89^ This method is expected to perform better than a vacuum HF ESP for molecules with highly extended π systems solvated by water, although it is also significantly more demanding to implement. Because FP chromophores are both less extended in their π systems and less solvent-exposed than the fluorophores considered by the Grubmüller group,^87^ we have utilized the standard RESP method here.

The RESP module within the AMBER suite^47^ was used to perform the fitting. Within the RESP method, we imposed three requirements consistent with the original Cornell charge model: (a) the total charge of all capping group atoms must be zero to allow them to be removed while maintaining an integer charge on the portion of the chromophore that will serve as the residue; (b) atomic charges of rotationally equivalent atoms within the molecule must be equal, with the exception of those found on capping groups; and (c) the atomic charges of the terminal/amide N, H, C, and O atoms in each chromophore must be consistent with the corresponding atoms in the standard ff19SB amino acids. For the DsRed and mCherry chromophores, there is no H on the amino terminus, so there does not exist a good model for the charge of the corresponding nitrogen. In these cases, we did not fix the charge of the N but instead fixed the charges of the C and O of the adjacent capping group to their Cornell equivalents, providing the nitrogen in question with neighboring atom charges that model a suitable MD environment, similar to the method described by Cieplak *et al*.^86^ Following those three constraints, the atomic charges were fit to provide the best possible reproduction of the QM-determined ESP. Further details are described in Results and Discussion.

### MD Simulation

MD simulations were executed on all six fluorescent proteins using the chromophore parameters reported here and starting from the fluorescent protein PDB structures given in Table 1. Histidines in EGFP (and related proteins EBFP, ECFP, and EYFP) were assigned to amino acid type HIE or HID based on assignments by previous researchers.^31^ Histidines in DsRed and mCherry were assigned to amino acid type HIE or HID using the H++ service.^90−92^ There is also a His within the EBFP chromophore itself. A review of EBFP literature left us unsure whether HID or HIE was the best assignment, so we chose to develop parameters for both possibilities. The “split the charge” method^93^ was used to determine how many chloride and sodium ions should be added to neutralize the solution while approximating 150 mM salt concentration using ions as described by Joung and Cheatham TIP4P/EW parameters.^94^ The system was solvated with OPC water^95,96^ in a truncated octahedron with a buffer size of 18 Å (roughly 16,000 water molecules).

Using AMBER,^47^ the system was minimized twice, each with 1000 steps. In the first minimization, the protein was held in place by a harmonic potential of 20 kcal/mol to allow the solvent to minimize around it; the second had no restraint. The system was then equilibrated to STP conditions using 10 stages of MD totaling 10 ns of simulation time. In the first stage, the system was heated using 500 ps of NVT MD with a harmonic potential of 20 kcal/mol on the protein and a 1 fs time step. Stages 2–9 comprised 1.9 total ns of NPT MD with 1 fs time steps over which the protein restraint was gradually reduced to 0 using an approximately logarithmic pattern and the pressure relaxation time was gradually increased from 1.0 to 20.0 ps. The final equilibration stage was 7.6 ns run using our production NPT MD conditions, which included the Langevin thermostat^97^ with a collision frequency of 5.0 ps^–1^, a pressure relaxation time of 20.0 ps, and a cutoff for van der Waals interactions of 10.0 Å. Particle Mesh-Ewald (PME) was utilized to handle long range electrostatic interactions.^98,99^ SHAKE was used to constrain all bonds containing hydrogen.^100^ The SHAKE and Ewald direct sum tolerances were set to 1 × 10^–6^. The random seed was set to a semirandom value every 10 ns based on the wall clock time.^101^ Total production simulation time was 1 μs for each protein.

Two production QM/MM simulations as implemented in AMBER^102^ were generated: one of EGFP and one of DsRed. The QM region was defined to be the one residue of the chromophore. The QM charge was set to −1 (see Table 1), and PM6 was utilized as the QM theory. QM/MM simulations utilized TIP-3P water with Joung and Cheatham TIP-3P Ewald ion parameters.^94^ Parameters were otherwise the same between the classical MM-only and QM/MM simulations. The total QM/MM production simulation time was 100 ns for each protein.

Simulation equilibration was confirmed using the RMSD of all atoms in the protein, the density, and also the total potential energy along with the van der Waals and electrostatic contributions. For all of these metrics, the standard deviation over the last 1 ns of equilibration was compared to the slope of a line fit to the same time span. The ratio of standard deviation to slope was greater than 50 for the RMSD and greater than 1000 for the density and the three energy-based metrics for all simulations. The stability of the protein was analyzed by plotting the proportion of residues in beta sheet conformation against time of the simulation. Decreasing numbers of residues in beta sheet conformation would indicate that the protein was unstable in the simulation and the beta barrel was degrading. The RMSD of just the chromophore atoms was used to analyze chromophore stability. Fluctuations in the chromophore structure were examined by looking at two dihedral angles between the two rings of the chromophores as well as a number of individual bond angles and dihedrals. Fluctuations in these angles and dihedrals were compared between the standard MD simulation and the QM/MM simulation to further evaluate the validity of MM parameters (see Results and Discussion). All analysis was completed using the cpptraj module from AmberTools.^103,104^

## Results and Discussion

### Atom Types

**Figure 4:**
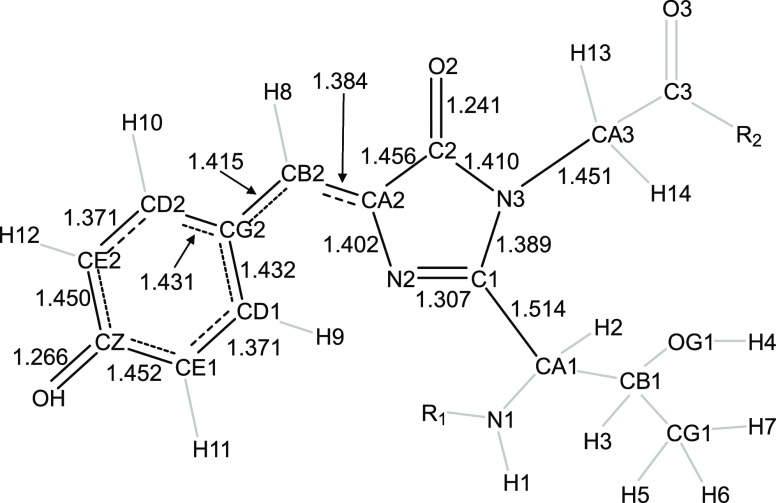
Atom naming convention for EGFP. Resonance is indicated with dashed bonds being more double and dotted more single in character. Bonds that were used for bond length comparison (*cf.*Table 4) are shown in black, whereas other bonds are in gray. Black bonds also have the B3LYP/6-31G(d)/SMD(1-pentanol) bond lengths indicated for reference. Similar figures with atom names for other chromophores are given in the Supporting Information.

For each fluorescent protein chromophore, some of the atoms share very similar chemical environments with the parent amino acid residues. In these cases, atom types from the standard parent residues in ff14SB have been used. Atoms whose bonding/environment have changed significantly include those in the newly formed five-membered imidazolidinone ring (e.g., C1, N2, CA2, C2, and N3 in EGFP; see Figures 3 and 4), the bridge between the two rings (e.g., CB2 and H8 in EGFP) and possibly the sidechain of the second amino acid (e.g., CG2, CD1, CD2, CE1, CE2, CZ, H9-12, and OH in EGFP). In particular, note that deprotonation of the tyrosine oxygen (in EGFP, EYFP, DsRed, and mCherry) is a key change because the CZ–OH bond has much more double bond character and the aromaticity of the phenyl ring is changed such that this sidechain no longer resembles tyrosine. This bonding character is illustrated with dashed and dotted bonds (along with the QM-optimized bond lengths) in Figure 4. For all of these conjugated atoms, several “systems” of parameters were considered. The standard ff14SB types (e.g., CA/CA for tyrosine), cc/cd (impure aromatic) from gaff, and ce/cf (conjugated) from gaff all contained reasonable chemical environment descriptions of these atoms. To quantify the differences in these descriptions, we looked in detail at all chromophores that share a tyrosine sidechain in this position (EGFP, EYFP, DsRed, mCherry), comparing the various bond length parameters to quantum mechanically optimized bond lengths using several different model chemistries as well as to the crystal structures. The overall RMSDs for these comparisons for EGFP are shown in Table 4. Similar tables for the other chromophores are available in the Supporting Information.

**Table 4:** RMSDs (in Å) of Bond Lengths of Just the Imidazolidinone, Bridging, and Tyrosine-Sidechain Portions of the Chromophore for EGFP^a^

	cc/cd bond parameters	CA/CA bond parameters	ce/cf bond parameters
crystal structure	**0.0454**	0.0537	0.0627
B3LYP/6-31G(d) SMD 1-pentanol	**0.0253**	0.0343	0.0318
B3LYP/6-31G(d) SMD water	**0.0236**	0.0341	0.0310
B3LYP/6-31G(d) vacuum	**0.0316**	0.0367	0.0360
mp2/cc-pvDZ PCM 1-pentanol	**0.0260**	0.0336	0.0325
mp2/cc-pvDZ PCM water	**0.0240**	0.0335	0.0312
mp2/cc-pvDZ vacuum	**0.0330**	0.0355	0.0376

a Three different force field parameter sets are compared to the crystal structure and to different QM-optimized structures. The smallest RMSD value in each row is in bold. RMSDs are calculated from the bonds shown in black in Figure 4.

For each of the seven reference geometries for the EGFP and EYFP chromophores, the cc/cd set of bond parameters gives the best comparison. The RMSDs given in Table 4 reflect all bonds within the conjugated portion of the chromophore (colored black and with bond lengths indicated in Figure 4). We also considered just the bonds of the imidazolidinone and bridging atoms separately from the tyrosine sidechain. For each of the individual units, the cc/cd bond parameters were also best. We also note that this structural comparison supported the carbonyl description of the CZ–OH bond; this carbon was represented as a carbonyl c rather than cc, and the oxygen was type o rather than oh. The bond length RMSDs for the three bonds of atom CZ were roughly a factor of 2 better with c/o atom types compared to cc/oh atom types.

**Figure 5:**
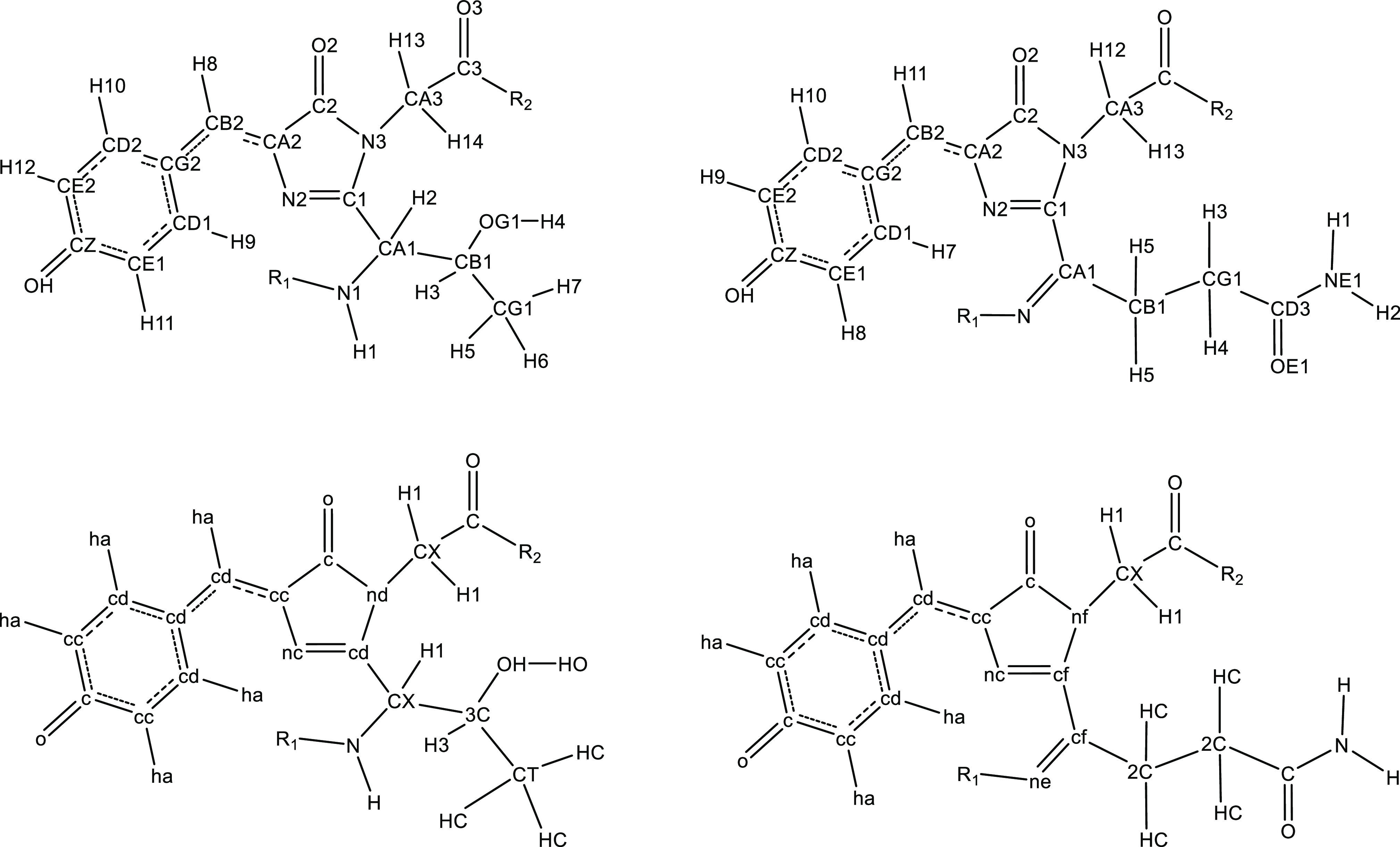
Final atom names (above) and types (below) for EGFP (left) and DsRed (right). Atom names and types for other chromophores are given in the Supporting Information.

Results with DsRed and mCherry similarly favored the cc/cd (and c/o) model for the tyrosine sidechain; however, the additional conjugated bonds extending into what had been the first amino acid were not well-described by cc/cd, so several options were explored for these bonds and the five-membered imidazolidinone ring. Using the same kind of comparison with QM structures, we found that atoms N, CA1, C1, and N3 were best described by ce/cf-style atom types, specifically ne, cf, cf, and nf for these four atoms. Figure 5 gives the final atom types for EGFP and DsRed. Analogous figures for all other chromophores are available in the Supporting Information.

EBFP and ECFP do not share the tyrosine sidechain of the other four chromophores, and so we again considered all of the bonding models. For all three chromophores (EBFP-HID, EBFP-HIE, and ECFP), the ff14SB parameter set and the cc/cd model from gaff yielded similar results for the portion of the chromophore that corresponds to this “second residue” sidechain. We calculated the RMSD between each of the two models and the QM-optimized bond lengths in the sidechain portions of the molecules. Averaging those RMSDs over all six QM geometries showed that the two models were different from each other by less than 0.005 Å within the sidechain portions of the molecules. In comparison, the ce/cf model was worse by >0.02 Å. Because these sidechains are essentially unchanged from their parent amino acids (in contrast to the deprotonated tyrosine in EGFP) and without a compelling structural reason to choose gaff over ff14SB, we have chosen to assign the standard ff14SB atom types. The imidazolidinone ring and bridging atoms are still best described by the cc/cd model and share those atom types with EGFP. Details for each of these models for the sidechain and imidazolidinone portions of these three chromophores are available in the Supporting Information.

Overall, we believe that our atom types yield a well-defined modular description of these fluorescent proteins. For the imidazolidinone ring and bridging atoms, we have developed two models: for chromophores sharing EGFP’s structure, we use the cc/cd style atom types from gaff, and for those with DsRed’s structure, we use a mixture of cc/cd and ce/cf atom types from gaff (Figure 5). For the sidechain of the “second residue”, chromophores with a deprotonated tyrosine use the cc/cd (and c/o) atom types from gaff as in EGFP. Chromophores with a sidechain that is essentially unchanged from the parent amino acid use standard Cornell-style atom types. In the end, this is a relatively simple model and provides a well-defined procedure (see Conclusions) for others to follow when applying these parameters to other fluorescent protein chromophores.

Because our descriptions of these xFP chromophores are consistent with ff14SB and ff19SB (and other Cornell-based force fields), we assume that the user will have loaded one of these complete force fields, and those values will be used without modification. Any other parameters must be explicitly defined within an AMBER force field modification (frcmod) file. We have developed a single frcmod file that contains parameters for all seven chromophores. This file is available through AmberTools and in the Supporting Information of this work. To help clarify the presentation of the results, we discuss each of the sections of the file (e.g., bond, angle, dihedral) separately, although they are all contained in a single frcmod file in AMBER.

### Bond Parameters

Table 5 gives the bond parameters from the frcmod file. Bonds 1–14 are direct matches for parameters that already exist in gaff, and those parameters were used without modification. Bonds 15–18 involve at least one gaff atom that is of the conjugated or improper aromatic type (cd, cf, nd, ne) for which there is no close analogy in ff14SB. Therefore, we chose to use gaff parameters for all of these, making the fairly straightforward substitutions of gaff tetrahedral c3 for tetrahedral ff14SB 2C and CX and gaff carbonyl c for ff14SB carbonyl C.

**Table 5:** Bond Parameters Beyond Those in ff14SB and ff19SB Needed for the Seven Chromophores

bond #	parameter	*K*_r_^a^	*r*_eq_^b^	source
direct matches for gaff parameters				
1	cc-cd	500.9	1.3729	gaff cc-cd
2	cd-cd	419.8	1.4278	gaff cd-cd
3	cc-nc	441.1	1.3694	gaff cc-nc
4	cd-nc	525.4	1.3172	gaff cd-nc
5	cd-nd	441.1	1.3694	gaff cd-nd
6	cc-c	371.0	1.4676	gaff c-cc
7	c-nd	416.9	1.3867	gaff c-nd
8	cc-ha	349.1	1.0837	gaff cc-ha
9	cd-ha	349.1	1.0837	gaff cd-ha
10	cf-cf	382.8	1.4574	gaff cf-cf
11	cf-ne	564.4	1.2964	gaff cf-ne
12	cf-nf	406.9	1.3942	gaff cf-nf
13	c-nf	411.2	1.3909	gaff c-nf
14	c-o	637.7	1.2183	gaff c-o
straightforward matches between gaff and ff14SB types				
15	cd-CX	334.8	1.5015	gaff c3-cd
16	nd-CX	334.7	1.4560	gaff c3-nd
17	cf-2C	320.9	1.5157	gaff c3-cf
18	ne-C	411.2	1.3909	gaff c-ne
parameters that treat atom CG2 as type cd				
19	cd-CC	419.8	1.4278	gaff cd-cd
20	cd-C*	419.8	1.4278	gaff cd-cd
special cases				
21	nc-cf	525.4	1.3172	gaff nc-cd
22	nf-CX	334.7	1.4560	gaff c3-nd

a Force constant of the bond potential in kcal/(mol·Å^2^).

b Equilibrium bond length in Å.

For ECFP and EBFP the atom that bridges between the imidazolidinone ring and the sidechain of the “second residue” (atom name CB2) must also bridge between the gaff cd atom type and a ff14SB atom type that is either C* (for ECFP) or CC (for EBFP). The same QM calculations that suggested using cc/cd types for the imidazolidinone ring also suggest that this bond most closely matches the gaff cd-cd type for both ECFP and EBFP. Therefore, the gaff cd-cd parameters are used in bond parameters 19–20.

The last two bond parameters (21–22) are special cases and require lengthier description, which is presented in the Supporting Information.

### Angle Parameters

As with bonds, many of the angle parameters are already defined within the existing ff14SB force field. The remaining angle parameters are given in Table 6. The first 17 are exact matches for existing gaff parameters, and the 18th involves the use of gaff type ne for nf. Angles 19–35 involve at least one gaff atom that is of the improper aromatic or conjugated type (cd, cf, nc, nd, ne, nf) for which there is no close analogy in ff14SB but for which there was a fairly clear substitute. For these, we used the central atom to dictate which parameter file to use as our source. Angles 19–22 have gaff central atoms, so we looked to gaff and used c3 in place of the tetrahedral CX and 2C types. Angles 23–35 have ff14SB central atoms; for all of these angles we substituted the sp^2^ C type for sp^2^ types cd and cf and, similarly, N for nd, ne, and nf.

**Table 6:** Angle Parameters Beyond Those in ff14SB and ff19SB Needed for the Seven Chromophores

angle #	parameter	*K*_θ_^a^	θ_eq_^b^	source
direct matches for gaff parameters				
1	cc-nc-cd	71.800	105.490	gaff cc-nc-cd
2	cc-c-cc	64.600	115.840	gaff cc-c-cc
3	cc-c-nd	67.800	116.240	gaff cc-c-nd
4	cc-c-nf	69.000	112.220	gaff cc-c-nf
5	cc-c-o	69.100	123.930	gaff cc-c-o
6	cc-cd-ha	48.500	121.760	gaff cc-cd-ha
7	cd-cd-cc	68.200	114.190	gaff cc-cd-cd
8	cd-cc-nc	72.200	111.650	gaff cd-cc-nc
9	cd-cd-ha	47.100	121.070	gaff cd-cd-ha
10	cd-cc-ha	48.500	121.760	gaff cd-cc-ha
11	nc-cd-nd	74.000	115.830	gaff nc-cd-nd
12	nd-c-o	73.900	123.180	gaff nd-c-o
13	nf-c-o	73.000	125.810	gaff nf-c-o
14	c-cc-cd	65.100	121.350	gaff c-cc-cd
15	c-cc-nc	66.200	123.320	gaff c-cc-nc
16	c-cc-ha	46.900	116.640	gaff c-cc-ha
17	cf-cf-ne	68.600	120.790	gaff cf-cf-ne
18	cf-cf-nf	68.600	120.790	gaff cf-cf-ne
straightforward matches between gaff and ff14SB types				
19	CX-cd-nd	66.000	120.950	gaff c3-cd-nd
20	CX-cd-nc	66.500	122.410	gaff c3-cd-nc
21	2C-cf-ne	66.900	120.680	gaff c3-cf-ne
22	2C-cf-cf	63.400	117.120	gaff c3-cf-cf
23	N-CX-cd	63.000	110.100	PARM10 C-CX-N
24	C-CX-nd	63.000	110.100	PARM10 C-CX-N
25	C-CX-nf	63.000	110.100	PARM10 C-CX-N
26	O-C-ne	80.000	122.900	PARM10 N-C-O
27	CX-C-ne	70.000	116.600	PARM10 CX-C-N
28	XC-C-ne	70.000	116.600	PARM19 XC-C-N
29	H1-CX-cd	50.000	109.500	PARM10 C-CX-H1
30	H1-CX-nd	50.000	109.500	PARM10 H1-CX-N
31	H1-CX-nf	50.000	109.500	PARM10 H1-CX-N
32	3C-CX-cd	63.000	111.100	ff14SB C-CX-3C
33	2C-CX-cd	63.000	111.100	ff14SB C-CX-2C
34	cf-2C-2C	63.000	111.100	ff14SB C-2C-2C
35	cf-2C-HC	50.000	109.500	ff14SB C-2C-HC
parameters that treat atom CG2 as type cd				
36	cc-cd-CC	68.200	114.190	gaff cc-cd-cd
37	CC-cd-ha	47.100	121.070	gaff cd-cd-ha
38	cc-cd-C*	68.200	114.190	gaff cc-cd-cd
39	C*-cd-ha	47.100	121.070	gaff cd-cd-ha
parameters that maintain ring behavior in Trp and His				
40	cd-C*-CB	70.00	128.60	PARM10 CT-C*-CB
41	cd-C*-CW	70.00	125.00	PARM10 CT-C*-CW
42	cd-CC-NA	70.00	120.00	PARM10 CT-CC-NA
43	cd-CC-CV	70.00	120.00	PARM10 CT-CC-CV
44	cd-CC-NB	70.00	120.00	PARM10 CT-CC-NB
45	cd-CC-CW	70.00	120.00	PARM10 CT-CC-CW
special cases				
46	cd-cd-cd	66.600	120.020	gaff ca-ca-ca
47	cd-nd-CX	66.700	120.490	gaff c-nd-cc
48	cd-nd-c	66.700	120.490	gaff c-nd-cc
49	c-nd-CX	66.700	120.490	gaff c-nd-cc
50	cf-nf-c	66.500	120.830	gaff c2-nf-ca
51	cf-nf-CX	66.500	120.830	gaff c2-nf-ca
52	c-nf-CX	66.500	120.830	gaff c2-nf-ca
53	cc-nc-cf	71.800	105.490	gaff cc-nc-cd
54	nf-cf-nc	70.200	127.960	gaff nf-cf-ne
55	cf-cf-nc	68.600	120.790	gaff cf-cf-ne
56	C-ne-cf	67.700	118.530	gaff c-ne-c2

a Force constant of the angular potential in kcal/(mol·radian^2^).

b Equilibrium angle in degrees.

Angles 36–39 have the bridging atom CB2 (type cd) as the central atom. One end-atom is either cc or ha from gaff; the other is part of the aromatic ring that is native to the second side chain and is type C* (for ECFP) or CC (for EBFP). Because the bond parameter in this location is modeled as cd-cd for all chromophores, the analogous substitution (either cd-cd-cc or cd-cd-ha) was made for these angle parameters.

For the adjacent angles within the aromatic rings of ECFP and EBFP (angles 40–45), the central atom is a special type (C* in Trp and CC in His) specific to that parent amino acid, so these unique ff14SB angle parameters were retained in the chromophores.

The remaining angle parameters (46–56) are special cases and require lengthier description, which is presented in the Supporting Information.

### Dihedral Parameters

Dihedral parameters are given in Table 7. The first 10 dihedrals are exact matches for existing gaff parameters. Dihedrals 11 and 12 describe the CB2-CG2 bond that is type cd-cd as described earlier; therefore, we assigned the gaff X-cd-cd-X parameter. Dihedrals 13–15 involve one ff14SB atom type and one gaff atom that is of the conjugated or impure aromatic type (cd, ne, nf) for which there is no close analogy in ff14SB. As with bonds and angles, we chose to use gaff parameters for these, making simple substitutions of gaff c for ff14SB C (#13) and gaff c3 for tetrahedral ff14SB type CX (#14–15).

**Table 7:** Dihedral Parameters Beyond Those in ff14SB and ff19SB Needed for the Seven Chromophores

dihed #	parameter	bond paths	*V*_n_/2^a^	γ^b^	*n*^c^	source
direct matches for gaff parameters						
1	X-cc-cd-X	4	16.0	180	2	gaff X-cc-cd-X
2	X-cc-nc-X	2	9.5	180	2	gaff X-cc-nc-X
3	X-cc-c-X	4	11.5	180	2	gaff X-cc-c-X
4	X-cd-cd-X	4	16.0	180	2	gaff X-cd-cd-X
5	X-cd-nc-X	2	9.5	180	2	gaff X-cd-nc-X
6	X-cd-nd-X	2	9.5	180	2	gaff X-cd-nd-X
7	X-c-nd-X	2	8.0	180	2	gaff X-c-nd-X
8	X-c-nf-X	2	0.4	180	2	gaff X-c-nf-X
9	X-cf-nf-X	2	1.6	180	2	gaff X-cf-nf-X
10	X-cf-cf-X	4	4.0	180	2	gaff X-cf-cf-X
parameters that treat atom CG2 as type cd						
11	X-cd-CC-X	4	16.0	180	2	gaff X-cd-cd-X
12	X-cd-C*-X	4	16.0	180	2	gaff X-cd-cd-X
straightforward matches between gaff and ff14SB types						
13	X-C-ne-X	2	0.4	180	2	gaff X-c-ne-X
14	X-CX-cd-X	6	0.0	0	3	gaff X-c3-cd-X
15	X-CX-nf-X	6	0.0	0	3	gaff X-c3-n2-X and X-c3-nf-X
special cases						
16	X-CX-nd-X	6	0.0	0	3	gaff X-c3-n2-X and X-c3-nf-X
17	X-2C-cf-X	6	0.0	0	3	gaff X-c3-ca-X and X-c3-cc-X
18	X-cf-nc-X	2	9.5	180	2	gaff X-cd-nc-X
19	X-cf-ne-X	2	8.3	180	2	gaff X-c2-ne-X and X-c2-n2-X
using ff14SB parameters for sidechain torsions						
20	cd-CX-3C-CT	1	0.112	0	–4	ff14SB C-CX-3C-CT
21	cd-CX-3C-CT	1	0.148	0	–3	component 2
22	cd-CX-3C-CT	1	0.289	180	–2	component 3
23	cd-CX-3C-CT	1	0.406	180	1	component 4
24	cd-CX-3C-OH	1	0.156	0	–4	ff14SB C-CX-3C-OH
25	cd-CX-3C-OH	1	0.315	0	–3	component 2
26	cd-CX-3C-OH	1	0.119	180	–2	component 3
27	cd-CX-3C-OH	1	0.697	180	1	component 4
28	cd-CX-2C-OH	1	0.129	0	–4	ff14SB C-CX-2C-OH
29	cd-CX-2C-OH	1	0.401	0	–3	component 2
30	cd-CX-2C-OH	1	0.218	180	-2	component 3
31	cd-CX-2C-OH	1	0.661	180	1	component 4
32	cf-2C-2C-HC	1	0.160	0	3	ff14SB CX-2C-2C-HC
33	cf-2C-2C-C	1	0.138	0	–4	ff14SB CX-2C-2C-C
34	cf-2C-2C-C	1	0.412	180	–3	component 2
35	cf-2C-2C-C	1	0.083	0	–2	component 3
36	cf-2C-2C-C	1	0.196	180	1	component 4
37	cf-2C-2C-S	1	0.028	0	–4	ff14SB CX-2C-2C-S
38	cf-2C-2C-S	1	0.016	0	–3	component 2
39	cf-2C-2C-S	1	0.245	0	–2	component 3
40	cf-2C-2C-S	1	0.417	0	1	component 4

a Magnitude of the torsional potential in kcal/mol.

b Phase offset in degrees.

c Periodicity of the torsion (unitless).

Dihedral parameters 16–19 are special cases requiring lengthier description and are presented in the Supporting Information.

The remaining dihedrals 20–40 all represent sidechain torsions that were refined in ff14SB. For all of these dihedrals, only the first atom of the four is a gaff atom type; the other three are unchanged including the two central atoms, so we have retained the ff14SB parameters. Dihedrals 20–27 describe the Thr in EGFP and ECFP. Dihedrals 28–31 describe the Ser in EBFP. Dihedrals 32–36 describe the Gln of DsRed, and dihedrals 37–40 describe the Met of mCherry.

### Improper Torsions

Improper dihedral parameters, which serve to maintain planar centers, were assigned when appropriate. Note that the third atom is the center atom for improper dihedrals in AMBER. In the ff14SB and related force fields, all impropers are defined with angles of 180° (i.e., planar). All impropers in ff14SB and gaff have one of just four different values for the barrier magnitude: 10.5 kcal/mol for carbonyls (and X-N2-CA-N2/X-n2-ca-n2) in both ff14SB and gaff, 1.1 kcal/mol for most center atoms of sp^2^ character in ff14SB and everything else in gaff, 1.0 kcal/mol for amide nitrogen and a few other sp^2^ nitrogen centers in ff14SB, and 0 for some rings in ff14SB. The impropers needed for the chromophores here (see Table 8) include one carbonyl (#1), nine that fit the general sp^2^ center description above (#2–10) and were assigned force constants of 1.1 kcal/mol, and three that match special impropers for His and Tyr (#11–13) and are also 1.1 kcal/mol.

**Table 8:** Improper Dihedral Parameters Beyond Those in ff14SB and ff19SB Needed for the Seven Chromophores

improp #	parameter	*V*_n_/2^a^	γ^b^	*n*^c^	source/comment
1	X-X-c-o	10.5	180	2	gaff X-X-c-o and PARM10 X-X-C-O
2	X-X-cc-ha	1.1	180	2	all gaff and most PARM10 with sp^2^ center atom
3	X-X-cd-ha	1.1	180	2	all gaff and most PARM10 with sp^2^ center atom
4	c-cd-nd-CX	1.1	180	2	all gaff and most PARM10 with sp^2^ center atom
5	c-cf-nf-CX	1.1	180	2	all gaff and most PARM10 with sp^2^ center atom
6	cf-ne-cf-2C	1.1	180	2	all gaff and most PARM10 with sp^2^ center atom
7	nd-nc-cd-CX	1.1	180	2	all gaff and most PARM10 with sp^2^ center atom
8	nf-nc-cf-cf	1.1	180	2	all gaff and most PARM10 with sp^2^ center atom
9	cd-cd-cd-cd	1.1	180	2	all gaff and most PARM10 with sp^2^ center atom
10	nc-c-cc-cd	1.1	180	2	all gaff and most PARM10 with sp^2^ center atom
11	NA-CV-CC-cd	1.1	180	2	PARM10 CT-CV-CC-NA
12	NB-CW-CC-cd	1.1	180	2	PARM10 CT-CW-CC-NB
13	CW-CB-C*-cd	1.1	180	2	PARM10 CB-CT-C*-CW

a Magnitude of the torsional potential in kcal/mol.

b Phase offset in degrees.

c Periodicity of the torsion (unitless).

### Nonbonded Parameters

The mass, polarizability, and van der Waals parameters for each atom type were set to the values of matching types from gaff, which are identical to analogous types in ff14SB (see Table 9).

**Table 9:** Nonbonded Parameters Beyond Those in ff14SB and ff19SB Needed for the Seven Chromophores

atom type	mass^a^	α^b^	R*^c^	ϵ^d^
nd	14.01	0.530	1.8240	0.1700
nc	14.01	0.530	1.8240	0.1700
ne	14.01	0.530	1.8240	0.1700
nf	14.01	0.530	1.8240	0.1700
cc	12.01	0.360	1.9080	0.0860
cd	12.01	0.360	1.9080	0.0860
cf	12.01	0.360	1.9080	0.0860
c	12.01	0.616	1.9080	0.0860
ha	1.008	0.135	1.4590	0.0150
o	16.00	0.434	1.6612	0.2100

a In g/mol.

b Polarizability in Å^3^.

c van der Waals radius in Å.

d van der Waals well depth in kcal/mol.

### Partial Charges

As described in Methods, RESP fits^21,84−86^ were used to determine the partial charges on each atom in each of the chromophores. Fits were performed with several levels of constraints for comparison. First, a RESP fit was executed with no constraints other than enforcing rotational equivalence of methylene and methyl hydrogens^84^ (e.g., H5, H6, H7 and H13, H14 in EGFP). The resulting atomic charges (referred to as the free fit model) are the best possible atom-centered fixed-charge representation of the quantum mechanically determined ESP that can be achieved with RESP. This free fit model served as a reference for how well the remaining fits matched the ESP. Second, a constraint was introduced forcing the total charge of the ACE and NME capping groups to be neutral. This model (which we call cap fix) represents the minimal constraints needed to produce a residue that is useful for MD.^21,86^ However, for all chromophores, the resulting charges of the N-terminal amide N–H and the C-terminal carbonyl C–O differed significantly from the standard Cornell-type charges for these atoms, so further constraints were needed. Consistent with the Cornell force field, a third RESP fit was performed (called amide fix) in which the capping groups were constrained to be neutral and the charges of the N- and C-termini (e.g., EGFP’s N1, H1, C3, and O3) were constrained to match the consensus charges of the corresponding atoms in the Cornell family of force fields.^21,86^ Note that Cornell defined three different sets of consensus charges: for neutral, positive, and negative residues. Although some of the full chromophores have neutral or negative charge, we have used the neutral consensus charges because the original N- and C-terminal residues were neutral for all FPs examined here. For DsRed and mCherry, there is no hydrogen on the N-terminus; as described in Methods, the charges of the carbonyl atoms in the adjacent ACE capping group were fixed to the ff14SB charges during fitting, providing a balanced charge environment for the N-terminal nitrogen, similar to the procedure described by Cieplak *et al*.^86^ Note that the resulting charges for that nitrogen in DsRed and mCherry are −0.292329 and −0.283649, respectively, similar to the −0.2548 used by Cornell for the nitrogen atom in proline that similarly lacks a hydrogen.

**Figure 6:**
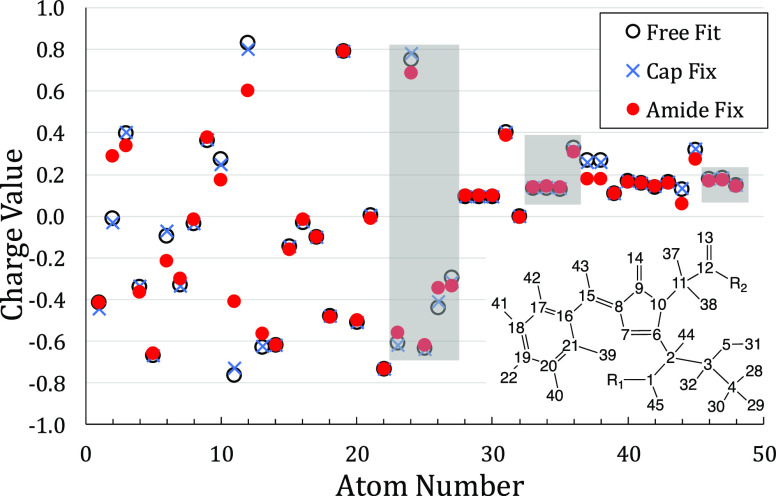
Partial charge models for the EGFP chromophore. The free fit model is the best atomic charge RESP representation of the quantum mechanical ESP. The cap fix model fixes the total of all capping group charges (atoms 23–27, 33–36, and 46–48; shaded gray) to be zero. The amide fix model also requires terminal N–H and C–O charges (atoms 1, 12, 13, and 45) to match corresponding residue charges in the ff14SB force field to enforce consistency with all other residues in the protein. Atom numbering given in the inset.

In the amide fix model, most chromophores had several atomic partial charges that differed by more than 0.10 charge units from their charges in the free fit model, as shown in Figure 6 for EGFP. In EGFP, there are four atoms with differences between their amide fix and free fit charges that exceed 0.10 charge units: atoms 2, 6, 11, and 12. The three largest differences are carbons 11, 2, and 12 (CA3, CA1, and C3; see Figure 5 for atom naming and the inset of Figure 6 for atom numbering), which are either one of the fixed atoms (C3) or adjacent to a fixed atom (CA3 and CA1). Note that atoms C3 and CA3 are the most positive (+0.830) and most negative (−0.768) atoms, respectively, in the molecule in the free fit model. Their charges are less extreme in the amide fix model, which likely lead to a more balanced MD behavior. The fourth atom is carbon C1, which is adjacent to CA1. Looking further, the atom with the fifth largest charge difference is #10 (nitrogen N3). These five atoms (2, 6, 10, 11, and 12) form a chain connecting the N and C termini of the chromophore, two of the atoms whose charges were fixed. In fact, every atom whose charge differs by more than 0.05 charge units between the free fit and amide fix models either is in a capping group or is within two atoms of the N and C terminal atoms. Thus, these charge discrepancies are unavoidable if the N and C terminal charges of the chromophore are to be balanced with those of the other residues in the protein.

Nonetheless, additional RESP fits were attempted in which the atom with the highest deviation in charge was fixed to its corresponding free fit value. This process did generally result in a reduced total RMS deviation in atomic charges compared to the free fit model. However, the quality of the charges compared directly to the QM-determined ESP generally got worse. This is because the partial charges of atoms adjacent to the newly fixed atom showed an increased deviation in comparison to the free fit. That is, fixing the charge of a “bad” atom did not actually solve the charge issue; it simply pushed the discrepancies onto different atoms in the chromophore.

In addition, fits were attempted with EGFP and mCherry using a five-amino-acid chromophore. The hope was that by providing more atoms in the system, charge discrepancies could be more widely spread, reducing the magnitude of individual atomic charge differences. In these five-residue systems, the three RESP fits described above were performed as well as four additional levels of constraints. None of these models yielded chromophore charges that were notably improved over the three-residue amide fix model. Again, regions of “bad” charge were simply shifted to different atoms, and the overall quality of fit was not improved.

**Figure 7:**
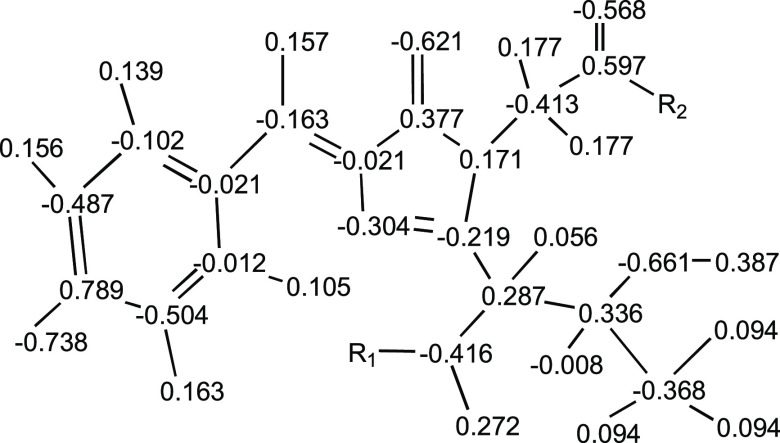
Final atomic charges for the EGFP chromophore. These match the amide fix model shown in Figure 6 and are given in tabular form in the Supporting Information for EGFP and all other chromophores.

Therefore, for all chromophores, we employed the three-residue amide fix model, which was the RESP fitting method with the fewest constraints that yielded charges consistent with the ff14SB force field. Final EGFP charges are shown in Figure 7 and are provided in tabular form in the Supporting Information along with similar charge tables for all chromophores.

### MD Simulation Results

When determining atom types and force field parameters, above, parameter values were chosen from existing force fields, with guidance from the QM-optimized geometry. This strong connection to the optimized geometry helps to ensure that the average structure of the chromophore during simulation reflects the optimized geometry. Drawing the parameters from the existing Cornell family of force fields, rather than, for instance, setting equilibrium values to match the QM geometry optimization or the crystal structure, accomplishes two goals: it provides a transferable parameter set that can be applied to multiple fluorescent protein chromophores, and it ensures that the dynamic behavior of the chromophore is well-balanced with the rest of the protein.

To provide a simple test of the validity of the parameters, MD simulations were executed. The results were examined with regard to (1) the overall stability of the protein and (2) the accuracy of the chromophore behavior.

The stability of the protein as a whole is likely not affected much by the parameters chosen to describe the chromophore and thus does not provide a very stringent test of the parameter validity. Nonetheless, we felt that it was important to verify that the protein is stable throughout a lengthy simulation. One microsecond of MD simulation was executed for each of the seven chromophores using explicit solvent as described in Methods. The 1-D and 2-D all-atom RMSD plots for EGFP (Supporting Information Figure S2) indicate that the protein continues to drift away from the crystal structure for approximately the first 400 ns. However, the structure then stabilizes, varying by less than 2 Å throughout the final 550 ns of simulation. Results for other proteins are similar, demonstrating that the protein structures as a whole are well-behaved within our simulations.

Because these proteins are made up of barrels of beta sheets, another measurement used to confirm protein stability was the number of residues in beta sheet conformation over the course of the simulation. The proteins were all generally stable throughout the simulation with most showing modest loss of beta sheet residues. For instance, a linear fit to the number of beta residues versus time for the EGFP simulation had a slope of −2.3 residues/μs. The crystal structure has 118 beta sheet residues, so this represents a loss of less than 2% over the full microsecond simulation. Note also that the standard deviation of beta sheet residues is 1.8 over the last 10 ns of the simulation (and also 1.8 over the last 1 ns of simulation), so the uncertainty in the number of beta sheet residues due to natural structural fluctuations of the protein is similar in size to the overall loss. The other proteins showed a similar behavior. EYFP had the largest loss with −3.2 residues/μs, and mCherry actually had a positive linear-fit slope of 0.72 residues/μs. The remaining proteins had slopes between −0.72 and −1.8 residues/μs. Standard deviations over 10 ns of simulation ranged from 0.96 to 1.8 residues with an average of 1.3 residues. Overall, the loss of beta sheet structure was slight, suggesting that the simulations are generally stable.

**Figure 8:**
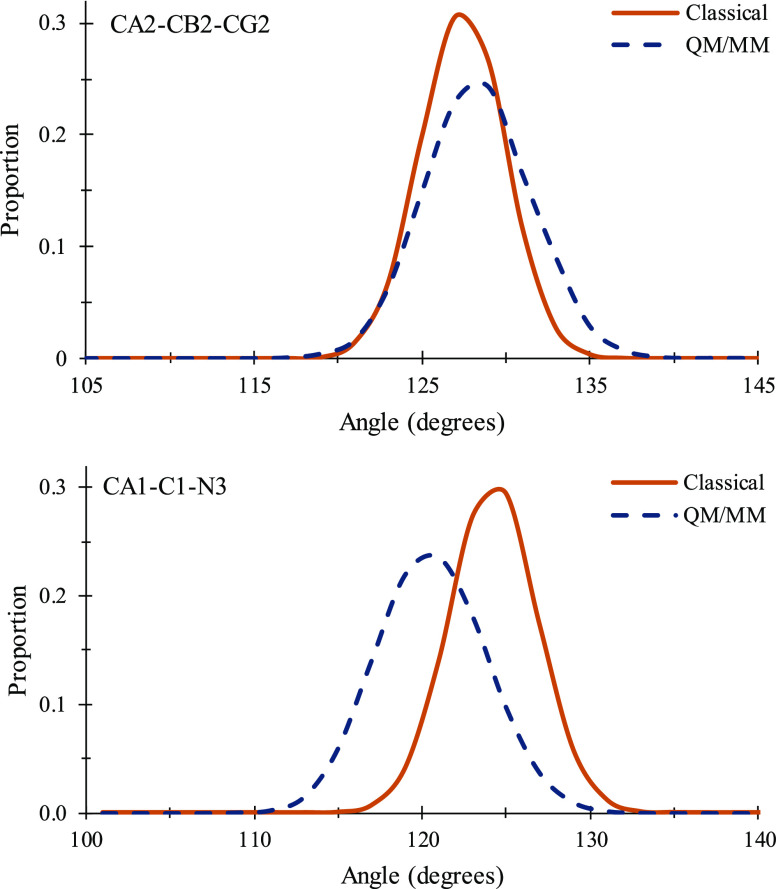
Histograms comparing distributions of two angles in the EGFP chromophore for both classical MD simulations using the parameters in this work and QM/MM simulations. The upper panel shows angle CA2-CB2-CG2, which is between the two conjugated rings. The lower panel shows CA1-C1-N3, which is the angle of what had been the alpha carbon of the first residue off the imidazolidinone ring. Both figures were made with 2° bin widths and have 40° *x*-axis ranges. See Figure 5 for atom naming.

**Figure 9:**
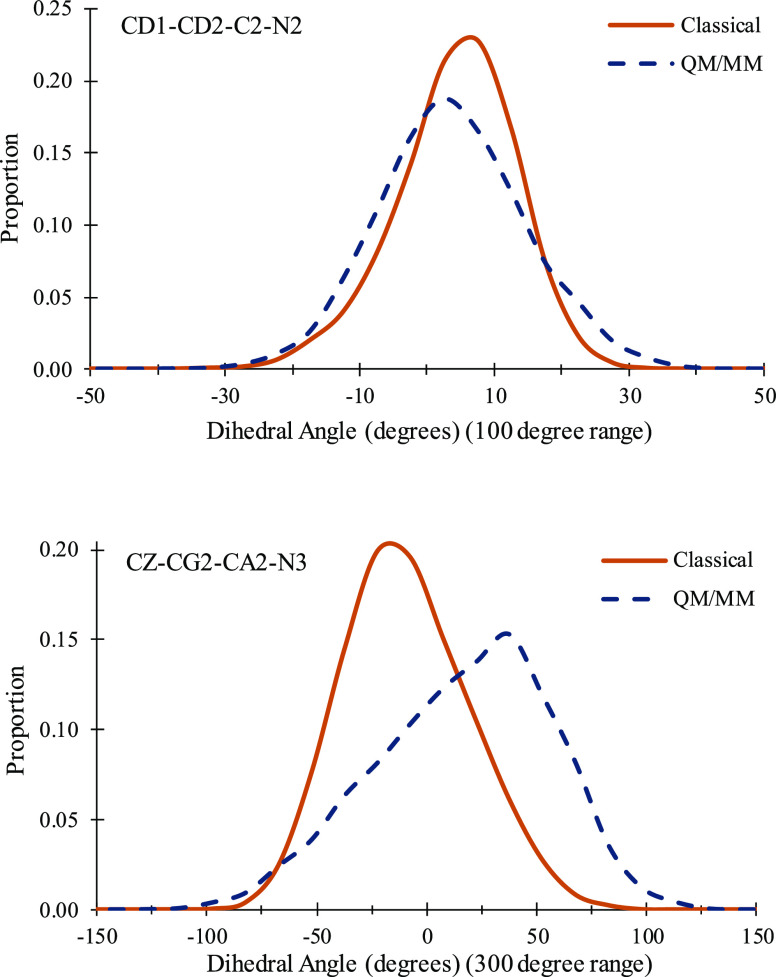
Histograms comparing distributions of two dihedral angles measuring the relative positions of the two conjugated rings in the EGFP chromophore. For each angle, comparison of classical MD simulations using the parameters in this work and QM/MM simulations is shown. The upper panel shows angle CD1-CD2-N2-C2 (created with 5° bin widths and a 100° *x*-axis range), and the lower panel shows CZ-CG2-CA2-N3 (created with 15° bin widths and a 300° *x*-axis range). See Figure 5 for atom naming.

Having verified the overall protein stability, we investigated the structures of the chromophores themselves in more detail. With the guidance of 2-D RMS analysis (plots not shown), we verified that the chromophores were stable, showing only modest sidechain rotations over the course of the simulations. However, visual inspection did reveal a modest deviation from planarity between the two rings of the chromophore. To examine this quantitatively, we ran 100 ns QM/MM simulations of EGFP and DsRed for comparison with the classical MD simulations as described in Methods. Two dihedral angles were defined to measure the planarity of the two rings: CD1-CD2-N2-C2 and CZ-CG2-CA2-N3. The distributions of these dihedrals as well as a variety of bond angles, bond-based dihedral angles, and improper dihedral angles were examined for the corresponding 100 ns of QM/MM and classical MD simulations. Angles to examine were focused on the imidazolidinone ring, the bridging bonds, and what had been the first amino acid in the chromophore yielding a set of 30 angles (14 dihedrals, 4 impropers, and 12 bond angles) for EGFP and a slightly different set of 30 angles (9 dihedrals, 3 impropers, and 18 bond angles) for DsRed. Plots showing histograms of the distributions of two of the bond angles and the two dihedrals that define planarity of the two rings are given in Figures 8 and 9, respectively, for EGFP. Similar plots of additional angles for EGFP and DsRed are given in the Supporting Information. Most of these angles yielded nearly Gaussian distributions, so to easily quantify the distributions in a way that minimized the impact of outlying values, a Gaussian curve was fit to each distribution, and its center and width (as measured by the standard deviation, σ) were used to characterize the distribution. Tables S2 and S5 in the Supporting Information give these values for EGFP and DsRed, along with the differences between the centers and the RMS of the widths.

Of the 60 angles examined, just two yielded center–center differences between the classical and QM/MM distributions that were more than 2 standard deviations apart. In fact, 47 of the centers were less than 1 standard deviation apart, and more than half, 31, were less than ^1^/_2_ standard deviation apart. Overall, the classical MD parameters gave very similar behavior to a simple QM description. One general difference between the classical and QM results is that the widths of the classical distributions were slightly narrower, on average 80% as wide (standard deviation 17%) as the QM/MM distributions. Just 3 of the 60 angles had classical distributions that were wider (all from DsRed: C1-CA1-N-C, N3-C1-CA1-N, and C^†^-N-CA1, where C^†^ is the carbonyl carbon of the next residue; see Table S5). This suggests that the classical force constants for the other 57 angles, which are mostly within the pi-conjugated portion of the chromophores, are generally too high, restricting motion. The angles that showed the largest deviation in means between the classical and QM/MM simulations were N-CA1-CB1 and N2-C1-N3 in DsRed (the two angles with center–center differences of more than 2σ); CB2-CA2-N2 in DsRed and N2-C1-CA1 in EGFP (two angles with center–center differences >5°); and N2-C1-CA1-N1 in EGFP, CB1-CG1-CD3-NE1 in DsRed, CA1-CB1-CG1-CD3 in DsRed, and the ring planarity dihedral CZ-CG2-CA2-N3 in both chromophores (five dihedral angles with center–center differences >15°). Of these nine angles, two are associated with the Gln sidechain in DsRed, which is entirely described by standard ff14/19SB atom types and parameters, and two are the ring dihedrals measuring long-range motion that is governed by a host of parameters, many of which lie outside the chromophore itself.

We believe the good overall agreement described above (and in Tables S2 and S5) validates the parameters presented in this work. Nonetheless, we could consider developing new values for some or all of the parameters needed to describe these chromophores to achieve stronger agreement between the classical and QM/MM simulations. However, we have taken great pains in our parameter selection process to maintain consistency and balance with the force field parameters used to describe every other residue in the simulation. Developing values that are optimized for just a few angles would lead to an imbalanced description of the chromophores. We could also consider developing an entirely customized parameter set for each of the chromophores, but that violates the desire to create a parameter set that is transferable to other FPs. Overall, we believe that the agreement is remarkably good between the QM/MM simulations and our classical descriptions of the chromophores using a highly transferable set of parameters that are easy to apply to other FP chromophores.

## Conclusions

In developing the parameters presented here, we sought to provide a foundation for future studies of other fluorescent protein chromophores. Thus, in addition to the parameters for these seven specific chromophores, we have also created a four-step procedure that can be broadly applied to yield balanced and consistent parameters for any fluorescent protein chromophore that shares the imidazolidinone ring of GFP.

1. Use the gaff-based atom types given here to describe the imidazolidinone ring of the chromophore, using either the EGFP/EYFP/ECFP/EBFP version or the extended DsRed/mCherry version, as appropriate (see Figure 5).
2. Consider the sidechain of what had been the second residue of the chromophore.
  a. If it is essentially unchanged from its original amino acid, use ff14/19SB atom types to describe it (e.g., EBFP and ECFP).
  b. If it is a deprotonated tyrosine, use the parameters given here for EGFP (e.g., EYFP, DsRed, mCherry).
  c. If it is something else, use gaff atom types to provide the best description, with guidance from quantum mechanical calculations of the equilibrium geometry.
3. The above atom types will define a list of parameters that are needed beyond those in ff14/19SB. Most, or perhaps all, of those parameters will already exist in the frcmod.xFPchromophores file that is part of the AmberTools distribution (also presented in Tables 5–9 this work and included in the Supporting Information). Any remaining parameters should be derived by analogy with parameters in frcmod.xFPchromophores, ff14/19SB, or gaff, again with guidance from quantum mechanical calculations.
4. Atomic charges should be calculated using RESP fitting consistent with the Cornell-type charges^21,86^ found in ff14/19SB. We’ve used the standard RESP procedure that involves finding a “good” molecular geometry, e.g., optimizing with B3LYP/6-31G(d) and SMD(1-pentanol), calculating an ESP with vacuum HF/6-31G(d), and performing a two-stage RESP fit using the amide fix procedure detailed in this work. Other options, such as QM/MM ensemble RESP fitting,^87^ can also be considered.

Using this procedure, we have developed a set of classical MD parameters describing chromophores from six common fluorescent proteins (with two versions of the EBFP chromophore). These parameters are drawn from and therefore fully compatible with the Cornell family of force fields,^21,44,45^ were developed in a modular fashion to provide maximum balance and consistency across the chromophores, and yield structural distributions that are similar to QM treatment of the chromophores, as judged by more than 7 μs of classical MD simulation and 200 ns of QM/MM simulation.

## Supporting Information Available

Additional Results section descriptions of the more challenging bond, angle, and dihedral parameters; comparison of PDB and optimized structures for each chromophore; RMSD plots from classical MD simulations for all FPs; atom names, types, and charges for each chromophore; comparison of different bond parameters for all chromophores aside from EGFP (similar to Table 4); and comparison of several angle and dihedral distributions from classical and QM/MM simulations for EGFP and DsRed (similar to Figures 8 and 9) (PDF)
The force field modification (frcmod) file needed to implement the parameters given here in AMBER (ZIP)
The library (lib) files needed to implement the parameters given here in AMBER (ZIP)
The command (leaprc) file that provides a convenient way to load them into AMBER’s system building tool, LEaP;^48^ the force field, library, and command files are also included in the free AmberTools package. In LEaP, after loading the desired force field (e.g., ff19SB), one can simply source the leaprc.xFPchromophores file to load all of the appropriate FP parameters. If the FP chromophore residue in the input PDB file has a three-letter code matching Table 1, then these parameters will be properly applied to the system (ZIP)

## Abbreviations

ACE: acetyl
CMAP: correction map
EBFP: enhanced blue fluorescent protein
ECFP: enhanced cyan fluorescent protein
EGFP: enhanced green fluorescent protein
ESP: electrostatic potential
EYFP: enhanced yellow fluorescent protein
FP: fluorescent protein
gaff: generalized amber force field
HF: Hartree–Fock
MD: molecular dynamics
MUE: mean unsigned error
NME: N-methyl amide
PDB: Protein Data Bank
QM: quantum mechanic(s/al/ally)
RESP: restrained electrostatic potential
RMS: root mean squared
RMSD: root mean squared deviation
