# Supplementary material for: Molecular Dynamics Force Field Parameters for the EGFP Chromophore and Some of Its Analogues
Source: J Phys Chem B. 2023 Jun 26;127(26):5772–88. doi: 10.1021/acs.jpcb.3c01486 (PMC10331734; doi:10.1021/acs.jpcb.3c01486)
Supplement: Supplementary file 1 — jp3c01486_si_001.pdf [file jp3c01486_si_001.pdf]

Supporting Information for

Molecular Dynamics Force Field Parameters for the EGFP Chromophore and Some of Its Analogues

Kimberly L. Breyfogle[#], Dalton L. Blood, Andreana M. Rosnik, and Brent P. Krueger*

Hope College Department of Chemistry, Holland, MI 49423

[#]Present Address: Kimberly L. Breyfogle, Department of Anthropology, Texas A&M University, College Station, TX, 77843, USA

* To whom correspondence should be addressed

This page left intentionally blank.

Contents of Supporting Information

Additional Results		S3
EGFP		
Figure S1:	Comparison of PDB and optimized structures for EGFP	S5
Figure S2:	1-D and 2-D RMSD plots for EGFP	S6
Table S1:	Atom name, charge, type for EGFP	S7
Figure S3:	Angle distribution plots for EGFP	S9
Figure S4:	Dihedral distribution plots for EGFP	S10
Table S2:	Classical and QM/MM angle and dihedral distribution stats for EGFP	S11
DsRed		
Figure S5:	Comparison of PDB and optimized structures for DsRed	S13
Figure S6:	1-D and 2-D RMSD plots for DsRed	S14
Table S3:	Atom name, charge, type for DsRed	S15
Table S4:	Parameter comparison table for DsRed	S17
Figure S7:	Angle distribution plots for DsRed	S18
Figure S8:	Dihedral distribution plots for DsRed	S19
Table S5:	Classical and QM/MM angle and dihedral distribution stats for DsRed	S20
EBFP - HID		
Figure S9:	Comparison of PDB and optimized structures for EBFP-HID	S22
Figure S10:	1-D and 2-D RMSD plots for EBFP (HID version)	S23
Figure S11:	Final atom names & atom types for EBFP (HID version)	S24
Table S6:	Atom name, charge, type for EBFP (HID version)	S25
Table S7:	Parameter comparison table for EBFP (HID version)	S27
EBFP - HIE		
Figure S12:	Comparison of PDB and optimized structures for EBFP-HIE	S29
Figure S13:	1-D and 2-D RMSD plots for EBFP (HIE version)	S30
Figure S14:	Final atom names and atom types for EBFP (HIE version)	S31
Table S8:	Atom name, charge, type for EBFP (HIE version)	S32
Table S9:	Parameter comparison table for EBFP (HIE version)	S34
ECFP		
Figure S15:	Comparison of PDB and optimized structures for ECFP	S36
Figure S16:	1-D and 2-D RMSD plots for ECFP	S37
Figure S17:	Final atom names and atom types for ECFP	S38
Table S10:	Atom name, charge, type for ECFP	S39
Table S11:	Parameter comparison table for ECFP	S41

EYFP

Figure S18:	Comparison of PDB and optimized structures for EYFP	S43
Figure S19:	1-D and 2-D RMSD plots for EYFP	S44
Figure S20:	Final atom names and atom types for EYFP	S45
Table S12:	Atom name, charge, type for EYFP	S46
Table S13:	Parameter comparison table for EYFP	S47

mCherry

Figure S21:	Comparison of PDB and optimized structures for mCherry	S48
Figure S22:	1-D and 2-D RMSD plots for mCherry	S49
Figure S23:	Final atom names and atom types for mCherry	S50
Table S14:	Atom name, charge, type for mCherry	S51
Table S15:	Parameter comparison table for mCherry	S53

Other files

Forcefield modification, library, and command files are also available for download

File 1:	frcmod.xFPchromophores.2022
File 2:	xFPchromophores.lib.2022
File 3:	leaprc.xFPchromophores

Additional Results

Bond Parameters

Bonds 1-20 are described in the main text. Bond 21 lies on a boundary between the cc/cd-type bonds in most of the chromophore conjugated system and the ce/cf-type bonds found in DsRed and mCherry. This particular bond is more double than single in character, so we considered three parameters from gaff: cd-nc, cd-ne, and cf-ne. The cd-nc parameter is the same one used in EGFP at this position and its bond length (1.317Å compared to 1.299Å and 1.296Å, respectively) compares best with the reference QM structures (average 1.335Å), so it was chosen. Bond 22 connects the nf-type nitrogen of the imidazolidinone ring to the alpha carbon of the 'third' amino acid in the chromophore. The obvious choice would be c3-nf, but this does not exist in gaff. EGFP uses c3-nd in this position, so that is the next most-likely candidate. Its bond length compares well with our QM calculations (c3-nd = 1.456Å and average QM = 1.452Å), so it was used here.

Angle Parameters

Angles 1-45 are described in the main text. Angle 46 (cd-cd-cd) has the atom named CG2 at the center and describes the connection between the deprotonated tyrosine and the bridging carbon. There actually is a cd-cd-cd parameter already in gaff, but it has an angle of 110.70°. The central atoms in this angle in the EGFP, EYFP, DsRed, and mCherry chromophores are sp2 in character and planar in all of our QM-optimized geometries. Thus, we sought a parameter with an angle of approximately 120° rather than the nearly-tetrahedral character of the existing cd-cd-cd. There were three angles in gaff that include three of the same sp2 carbon and have angles within a few degrees of 120°: c2-c2-c2 (121.81° with force constant 69.3 kcal/(mol·radian2)), ca-ca-ca (120.02°, 66.6 kcal/(mol·radian2)), and ce-ce-ce (122.11°, 63.4 kcal/(mol·radian2)). We selected the ca-ca-ca parameter, because it had the angle nearest to 120° (and the force constants for all three are similar).

Angles 47-49 describe the connection between the imidazolidinone ring and the alpha carbon of the 'third' amino acid of the chromophore in EGFP, EYFP, EBFP, and ECFP. The central atom here (named N3) is sp^2 in character and has a trigonal planar geometry. Of all the angles in gaff that have nd as the center atom bonded to any two types of carbons, there are only three with parameters closer to planar than to tetrahedral: cc-nd-cc (117.30°, 69.5 kcal/(mol·radian^2)), c-nd-cc (120.49°, 66.7 kcal/(mol·radian^2)), and c-nd-ca (120.66°, 65.9 kcal/(mol·radian^2)). We chose c-nd-cc for all three of these angles since it is the closest to 120°, and again the force constants provide no compelling differentiation.

Angles 50-52 describe the same location with N3 as the central atom for the DsRed and mCherry chromophores where it is type nf. Again, atom N3 is sp^2 in character and trigonal planar in geometry. Searching gaff for angles with nf between any two types of carbon yields six with angles closer to planar than to tetrahedral (and less than 125°). Only one of these is within

one degree of 120°, so for all three of these parameters we chose c2-nf-ca (120.83°, 66.5 kcal/(mol·radian^2)).

Angles 53-55 transition between the cc/cd-type bonds in most of the chromophore conjugated system and the ce/cf-type bonds found in DsRed and mCherry. While gaff did not contain parameters for any of these three angles, there were quite a few to choose from that maintained the type of the central atom with similar atoms on each end. We restricted ourselves to parameters that maintain the cc/cd or ce/cf style of the central atom, the elements of the three atoms, and the single/double bond pattern. For example, for cc-nc-cf (angle 53) the central atom is of cc/cd style with two carbons surrounding a central nitrogen via one single bond and one double bond. This suggests use of the cc-nc-cd parameter, which does exist in gaff, so that is what we selected. Similarly, nf-cf-nc (angle 54) leads to nf-cf-ne, and cf-cf-nc (angle 55) leads to cf-cf-ne.

For the final angle (56), substitution of gaff carbonyl c for ff14SB carbonyl C is straightforward, but the resulting c-ne-cf does not exist in gaff. There are three angles that maintain two of the three atom types: c-ne-c2, c-ne-cu, and ca-ne-cf. All three have similar parameters with force constants of 68 ± 1 kcal/(mol·radian^2) and angles of 120 ± 2°. We chose to use c-ne-c2 which has a more general atom type (c2 for cf) for the atom that doesn't match, rather than a more specific atom type (cu for cf or ca for c).

Dihedral Parameters

Dihedrals 1-15 and 20-40 are described in the main text. Dihedral 16, like 14 and 15, also involves substitution of c3 for CX, but the resulting X-c3-nd-X does not exist in gaff. There are two similar parameters that do exist in gaff, with either n2 or nf in place of nd. These both have the same dihedral parameters, which we have used here. Similarly, dihedral 17 involves substituting gaff c3 for the tetrahedral ff14SB 2C and use of ca or cc in place of cf. Dihedral 18 (X-cf-nc-X) spans the boundary between cc/cd and ce/cf types of bonding in DsRed and mCherry and represents a bond that is more double in character than single. This could be represented in gaff by either X-cf-ne-X or X-cd-nc-X. The first does not exist in gaff, but the second does, so we use X-cd-nc-X for this dihedral. While the cf-ne bond and multiple X-cf-ne and cf-ne-X angles exist in gaff, the X-cf-ne-X dihedral (#19) does not. We considered all gaff dihedrals with sp^2 carbon bonded to type ne or sp^2 nitrogen bonded to type cf. There were five of these, X-cf-nf-X, X-c-ne-X, X-c2-ne-X, X-ce-ne-X, and X-ca-ne-X. Of these five, two specifically represent conjugated systems with bonds that are more single in character (X-cf-nf-X and X-ce-ne-X) and two have barrier magnitudes of 0.4 kcal/mol (X-c-ne-X) and 0 kcal/mol (X-ca-ne-X) similar to dihedrals that represent single bonds. Because dihedral 19 is more double in character, we selected the remaining X-c2-ne-X. This choice is supported by the identical parameters (barrier magnitude of 8.3 kcal/mol, 180° phase, and 2-fold periodicity) of the more general carbon-nitrogen double bond of X-c2-n2-X.

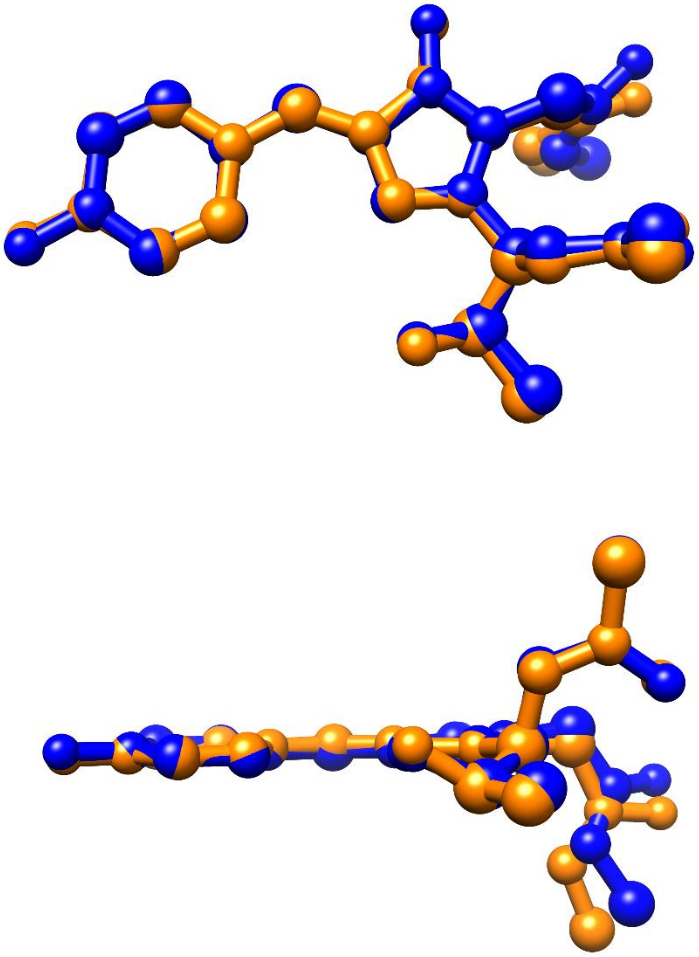

Figure S1. Comparison of PDB and quantum mechanically optimized structures for the EGFP chromophore. The upper and lower images are the same except they are rotated roughly 90 degrees. The PDB structure is in blue and the optimized structure in orange. A ball and stick representation was used where the atoms (balls) are scaled to 25% of the Van der Walls size and the bonds (sticks) are cylinders with 0.2 Å radii.

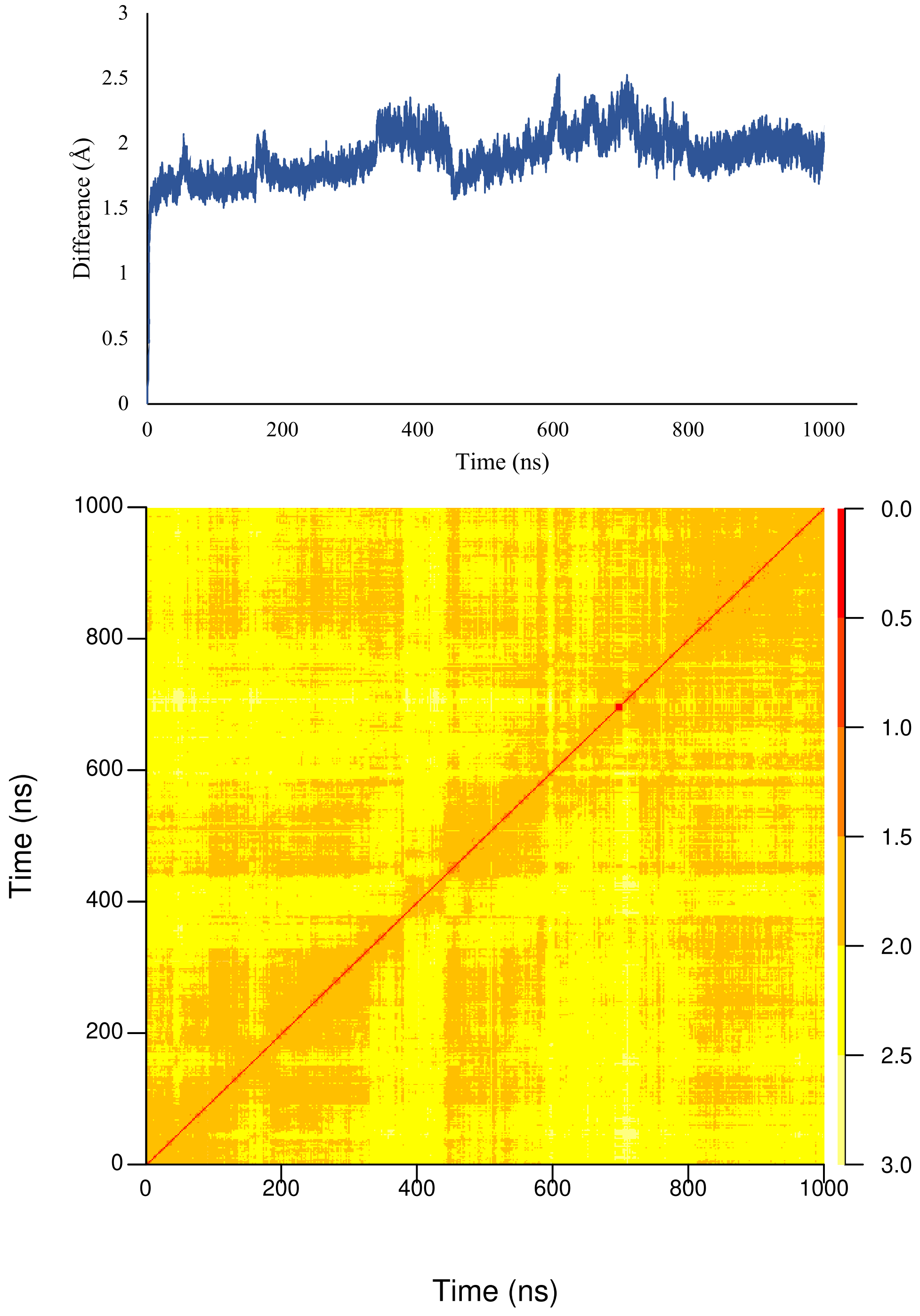

Figure S2. All-atom RMSD plots of EGFP: 1-D on top and 2-D on the bottom. The 1-D RMS is determined relative to the minimized crystal structure and includes equilibration. The first frame of the 2-D RMS is after 10 ns of equilibration.

Table S1. Atom names, types, and charges for the EGFP chromophore (PDB residue CRO).

Atom Name	Atom Type	Charge
CD2	cd	-0.101929
CE2	cc	-0.486564
CZ	c	0.789243
CG2	cd	-0.021390
CD1	cd	-0.011925
CE1	cc	-0.504196
CB2	cd	-0.163396
CA2	cc	-0.020935
C2	c	0.377080
H10	ha	0.138512
H12	ha	0.156037
H11	ha	0.162759
H9	ha	0.104630
H8	ha	0.156927
OH	o	-0.737525
O2	o	-0.620693
N2	nc	-0.304278
N3	nd	0.170822
C1	cd	-0.219250
CA3	CX	-0.412864
CA1	CX	0.287050
H13	H1	0.176453
H14	H1	0.176453
C3	C	0.597300
O3	O	-0.567900
N1	N	-0.415700

H1	H	0.271900
H2	H1	0.055481
CB1	3C	0.336064
CG1	CT	-0.367704
H5	HC	0.093660
H6	HC	0.093660
H7	HC	0.093660
H3	H1	-0.007665
OG1	OH	-0.660917
H4	HO	0.387141

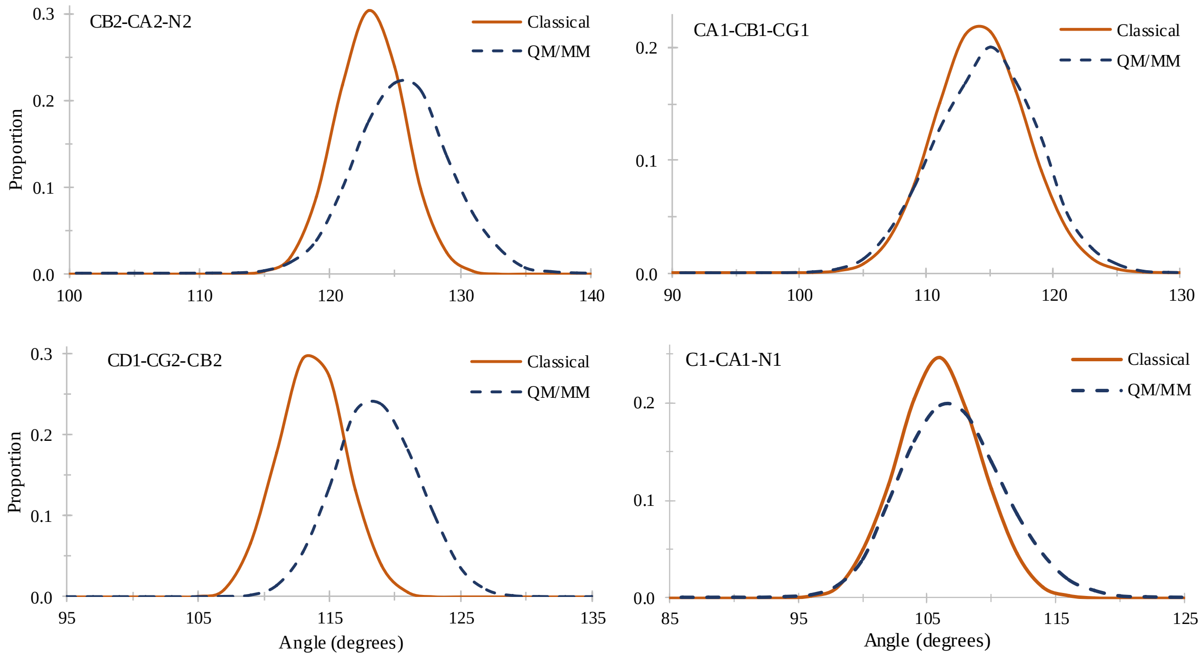

Figure S3. Histograms comparing distributions of four angles in the EGFP chromophore for both classical MD simulations using the parameters in this work and QM/MM simulations. The angle is identified in each panel. All figures were made using two-degree bin widths and 40-degree x-axis ranges. See Fig 5 for atom naming.

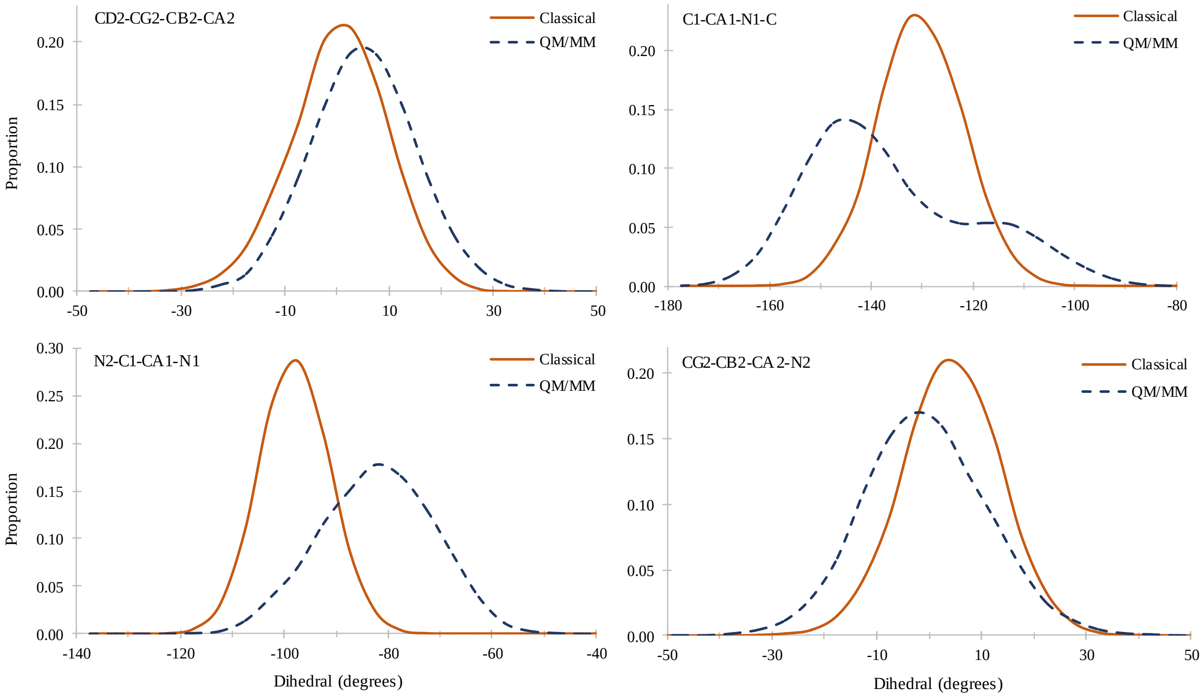

Figure S4. Histograms comparing distributions of four dihedral angles in the EGFP chromophore for both classical MD simulations using the parameters in this work and QM/MM simulations. The dihedral is identified in each panel. All figures were made using five-degree bin widths and with 100-degree x-axis ranges. See Fig 5 for atom naming.

Table S2. EGFP comparison of classical and QM/MM angle/dihedral distributions. All values are given in degrees. Similarity indicates how closely matching the centers of the two distributions are relative to the RMS of the sigmas. *** indicates the distributions are highly similar, with the difference in centers < $\sigma_{RMS}/2$. ** indicates center difference < σ_{RMS} and * indicates center difference < $2\sigma_{RMS}$. A blank entry indicates center difference > $2\sigma_{RMS}$.

Atoms in Angle or Dihedral	Classical Center	Classical Sigma	QM/MM Center	QM/MM Sigma	Center Difference	RMS of Sigmas	Similarity
CD1-CD2-N2-C2	9	14	5	18	3	16	***
CZ-CG2-CA2-N3	-12	29	22	41	-34	36	**
C1-CA1-N1-C†	-130.4	8.6	-140.6	16	10.1	12.8	**
C1-N3-C2-CA2	0.2	4.2	-4.4	6.0	4.6	5.2	**
C1-N3-CA3-C3	97.4	7.0	101.4	12.3	-4.0	10.0	***
C2-N3-C1-N2	-0.3	4.9	4.1	6.0	-4.4	5.5	**
CD2-CG2-CB2-CA2	0.8	9.2	5.1	10.0	-4.2	9.6	***
CE1-CD1-CG2-CD2	0.8	6.2	-0.4	7.9	1.3	7.1	***
CE2-CZ-CE1-CD1	-1.1	6.3	-2.6	8.8	1.5	7.6	***
CG2-CB2-CA2-N2	4.4	9.3	-1.1	11.7	5.6	10.6	**
CZ-CE1-CD1-CG2	0.2	6.6	1.9	7.6	-1.7	7.1	***
N2-C1-CA1-N1	-98.0	6.7	-81.8	11.3	-16.2	9.3	*
N2-CA2-C2-N3	-0.1	4.6	3.3	5.9	-3.4	5.3	**
N3-C1-N2-CA2	0.2	4.9	-1.9	5.3	2.1	5.1	***
OH-CE1-CZ-CE2	180.8	5.5	179.0	6.1	1.8	5.8	***
O2-CA2-C2-N3	178.2	5.3	180.2	7.6	-2.0	6.6	***
CA3-C1-N3-C2	183.3	6.8	195.7	12.6	-12.4	10.2	*
CA1-N2-C1-N3	182.3	6.5	181.5	7.1	0.8	6.8	***
C1-CA1-N1	106.9	3.2	107.8	3.9	-0.8	3.6	***
C1-N3-CA3	126.1	2.6	125.4	3.5	0.7	3.1	***
C2-N3-C1	109.3	2.1	107.2	2.2	2.1	2.1	**

CA1-C1-N3	124.2	2.6	128.8	3.5	-4.5	3.0	*
CA1-CB1-CG1	113.8	3.7	114.8	4.1	-1.0	3.9	***
CB2-CA2-C2	123.9	2.6	124.8	3.6	-0.9	3.2	***
CB2-CA2-N2	123.1	2.6	125.6	3.5	-2.4	3.1	**
CD1-CG2-CB2	113.7	2.5	118.5	3.2	-4.8	2.9	*
CG2-CB2-CA2	127.5	2.5	128.3	3.2	-0.8	2.9	***
CG2-CB2-H8	116.5	3.3	116.9	4.0	-0.4	3.7	***
N2-C1-CA1	123.3	2.6	128.8	3.5	-5.5	3.1	*
N2-C1-N3	112.1	2.2	110.2	2.2	1.9	2.2	**

† This carbon is the amide C of the next residue, bound to atom N1.

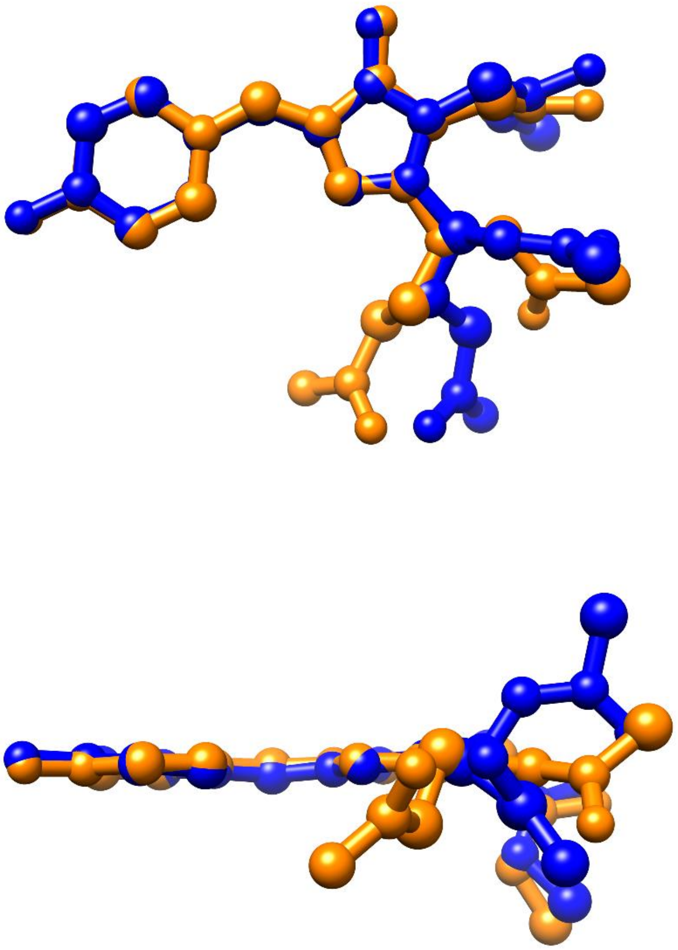

Figure S5. Comparison of PDB and quantum mechanically optimized structures for the DsRed chromophore. The upper and lower images are the same except they are rotated roughly 90 degrees. The PDB structure is in blue and the optimized structure in orange. A ball and stick representation was used where the atoms (balls) are scaled to 25% of the Van der Walls size and the bonds (sticks) are cylinders with 0.2 Å radii.

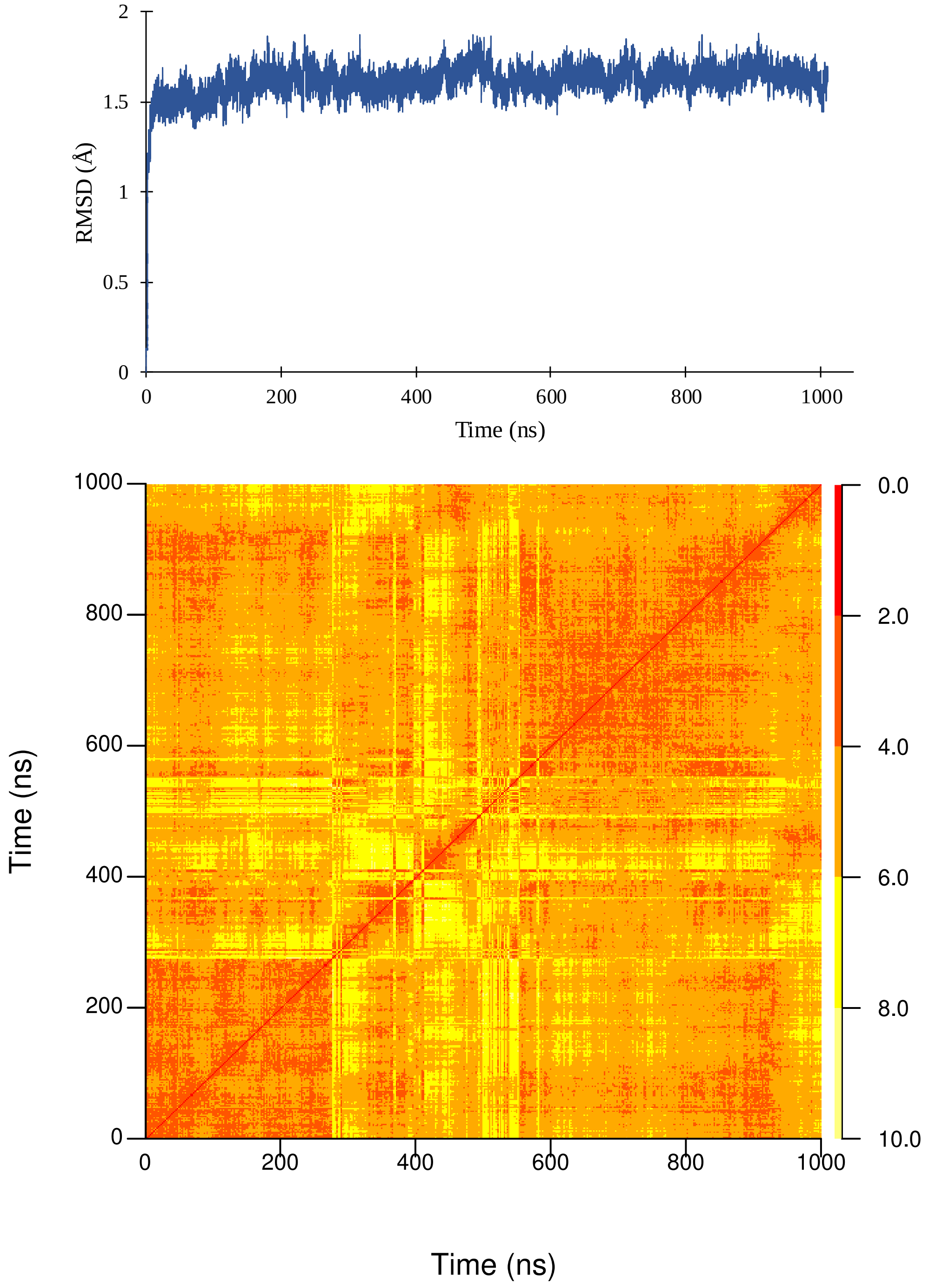

Figure S6. All-atom RMSD plots of DsRed: 1-D on top and 2-D on the bottom. The 1-D RMS is determined relative to the minimized crystal structure and includes equilibration. The first frame of the 2-D RMS is after 10 ns of equilibration.

Table S3. Atom names, types, and charges for the DsRed chromophore (PDB residue CRQ).

Atom Name	Atom Type	Charge
N	ne	-0.292329
CA1	cf	0.207110
CB1	2C	-0.066022
CG1	2C	-0.254932
CD3	C	0.917072
NE1	N	-1.097361
H1	H	0.446385
H2	H	0.446385
OE1	O	-0.664061
H3	HC	0.088826
H4	HC	0.088826
H5	HC	0.063263
H6	HC	0.063263
C1	cf	0.020610
N2	nc	-0.308148
CA2	cc	-0.069434
CB2	cd	-0.131918
CG2	cd	-0.011006
CD2	cd	-0.109773
CE2	cc	-0.470355
CZ	c	0.799172
CE1	cc	-0.504033
CD1	cd	-0.014350
H7	ha	0.128632
H8	ha	0.165022
OH	o	-0.718060

H9	ha	0.158116
H10	ha	0.141742
H11	ha	0.158757
C2	c	0.441755
O2	o	-0.657912
N3	nf	0.105614
CA3	CX	-0.434338
C	C	0.597300
O	O	-0.567900
H12	H1	0.167042
H13	H1	0.167042

Table S4. RMSDs (in Å) of bond lengths of just the imidazolidinone, bridging, and tyrosine-sidechain portions of the chromophore for DsRed. Three different force-field parameter sets are compared to the crystal structure and to different QM-optimized structures. The smallest RMSD value in each row is bold.

	cc/cd bond parameters	CA/CA bond parameters	ce/cf bond parameters
Crystal structure	**0.0461**	0.0492	0.0634
B3LYP/6-31G(d) SMD 1-pentanol	**0.0275**	0.0348	0.0331
B3LYP/6-31G(d) SMD water	**0.0262**	0.0343	0.0323
B3LYP/6-31G(d) vacuum	**0.0327**	0.0356	0.0380
mp2/cc-pvDZ PCM 1-pentanol	**0.0288**	0.0332	0.0347
mp2/cc-pvDZ PCM water	**0.0272**	0.0328	0.0336
mp2/cc-pvDZ vacuum	**0.0345**	**0.0345**	0.0410

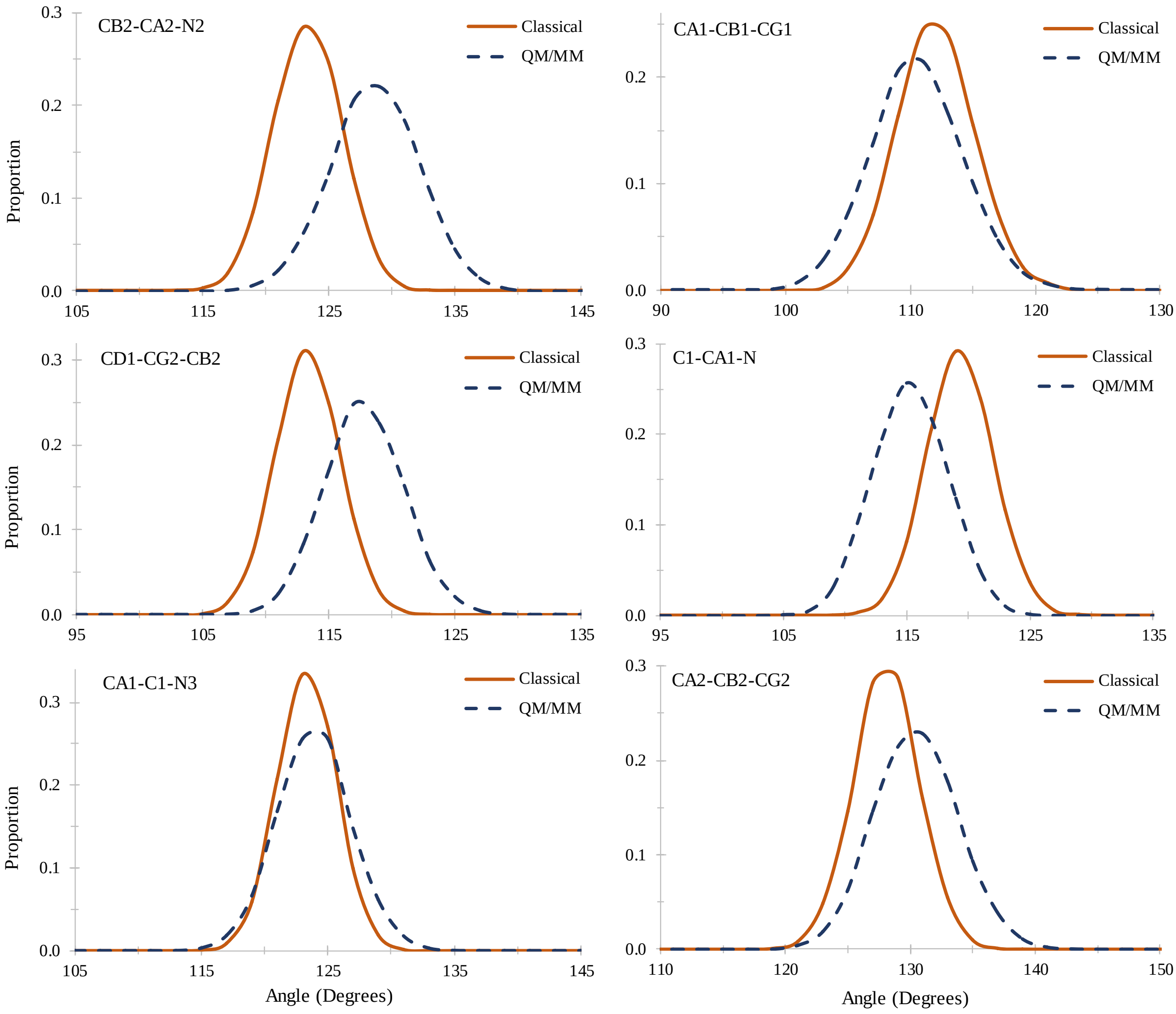

Figure S7. Histograms comparing distributions of six angles in the DsRed chromophore for both classical MD simulations using the parameters in this work and QM/MM simulations. The angle is identified in each panel. All figures were made using two degree bin widths. See Fig 5 for atom naming. Each figure has an x-axis range of 40 degrees, but the center of that range varies. The y-axis range also varies from figure to figure.

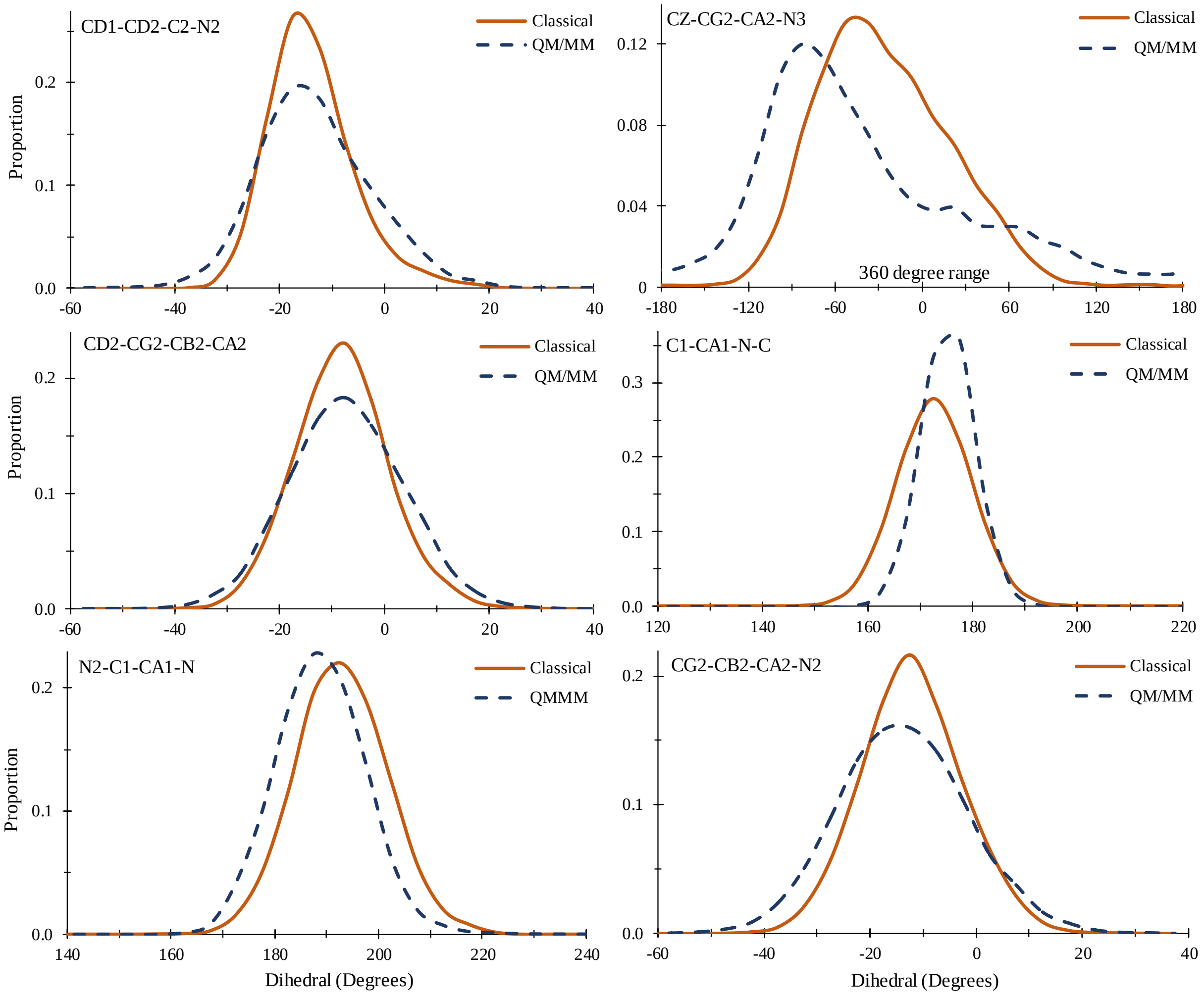

Figure S8. Histograms comparing distributions of six dihedral angles in the DsRed chromophore for both classical MD simulations using the parameters in this work and QM/MM simulations. The angle is identified in each panel. All figures were made using five-degree bin widths except CZ-CG2-CA2-N3, which used 15-degree bins. See Fig 5 for atom naming. Each figure has an x-axis range of 100 degrees except CZ-CG2-CA2-N3, which has a 360-degree range. The y-axis range varies from figure to figure.

Table S5. DsRed comparison of classical and QM/MM angle/dihedral distributions. All values are given in degrees. Similarity indicates how closely matching the centers of the two distributions are relative to the RMS of the sigmas. *** indicates the distributions are highly similar, with the difference in centers < $\sigma_{RMS}/2$. ** indicates center difference < σ_{RMS} and * indicates center difference < $2\sigma_{RMS}$. A blank entry indicates center difference > $2\sigma_{RMS}$.

Atoms in Angle or Dihedral	Classical Center	Classical Sigma	QM/MM Center	QM/MM Sigma	Center Difference	RMS of Std Devs	Similarity
CD1-CD2-C2-N2	156.0	11.1	158.2	14.1	-2.1	12.7	***
CZ-CG2-CA2-N3	-32	46	-65	57	33	52	**
C1-CA1-N-C	172.6	7.0	175.4	5.0	-2.7	6.1	***
CA1-CB1-CG1-CD3	137.7	10.8	121.5	13.7	16.2	12.3	*
CB1-CG1-CD3-NE1	-155	14	-133	29	-23	23	**
CD2-CG2-CB2-CA2	-8.4	8.7	-7.4	10.9	-0.9	9.9	***
CG2-CB2-CA2-N2	-12.4	9.4	-14.2	12.0	1.8	10.8	***
N-CA1-CB1-CG1	-79.9	8.6	-82.3	10.4	2.4	9.5	***
N3-C1-CA1-N	7.5	9.9	4.5	9.7	2.9	9.8	***
CA1-N2-C1-N3	180.5	0.4	183.7	6.6	-3.2	4.7	**
CA3-C1-N3-C2	-170.1	9.4	-160.5	12.0	-9.6	10.8	**
O2-CA2-C2-N3	181.2	6.2	181.1	6.9	0.1	6.5	***
C†-N-CA1	126.2	3.1	122.1	2.8	4.1	2.9	*
C1-CA1-CB1	119.2	2.5	115.0	3.1	4.2	2.8	*
CA1-C1-N2	121.0	2.5	125.2	3.1	-4.2	2.8	*
C1-CA1-N	119.3	2.7	115.3	3.1	4.0	2.9	*
C1-N3-CA3	130.2	2.6	129.8	3.4	0.3	3.0	***
C2-N3-C1	107.3	2.1	106.5	2.2	0.8	2.2	***
C2-N3-CA3	121.2	2.5	119.9	3.3	1.3	2.9	***

CA1-C1-N3	123.4	2.3	123.9	2.9	-0.5	2.6	***
CA1-CB1-CG1	112.0	3.1	110.4	3.6	1.6	3.4	***
CA2-CB2-H11	115.7	3.3	113.1	3.9	2.6	3.6	**
CA2-CB2-CG2	128.1	2.6	130.5	3.4	-2.4	3.0	**
CB1-CG1-CD3	114.9	3.0	111.7	3.7	3.2	3.4	**
CB2-CA2-C2	121.0	2.7	121.0	3.4	0.0	3.1	***
CB2-CA2-N2	123.3	2.7	128.7	3.5	-5.4	3.1	*
CD2-CG2-CB2	125.3	2.5	124.5	3.2	0.8	2.9	***
CG2-CB2-H11	115.8	3.3	116.0	4.0	-0.2	3.7	***
N-CA1-CB1	120.8	2.5	129.3	3.2	-8.4	2.9	
N2-C1-N3	115.1	2.2	110.4	2.2	4.8	2.2	

† This carbon is the amide C of the next residue, bound to atom N.

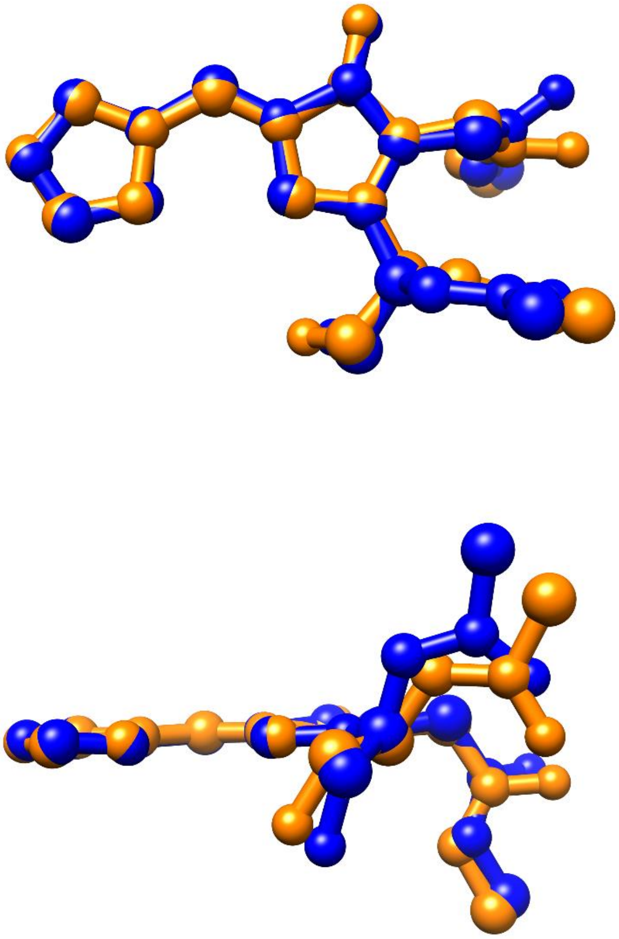

Figure S9. Comparison of PDB and quantum mechanically optimized structures for the HID form of the EBFP chromophore. The upper and lower images are the same except they are rotated roughly 90 degrees. The PDB structure is in blue and the optimized structure in orange. A ball and stick representation was used where the atoms (balls) are scaled to 25% of the Van der Walls size and the bonds (sticks) are cylinders with 0.2 Å radii.

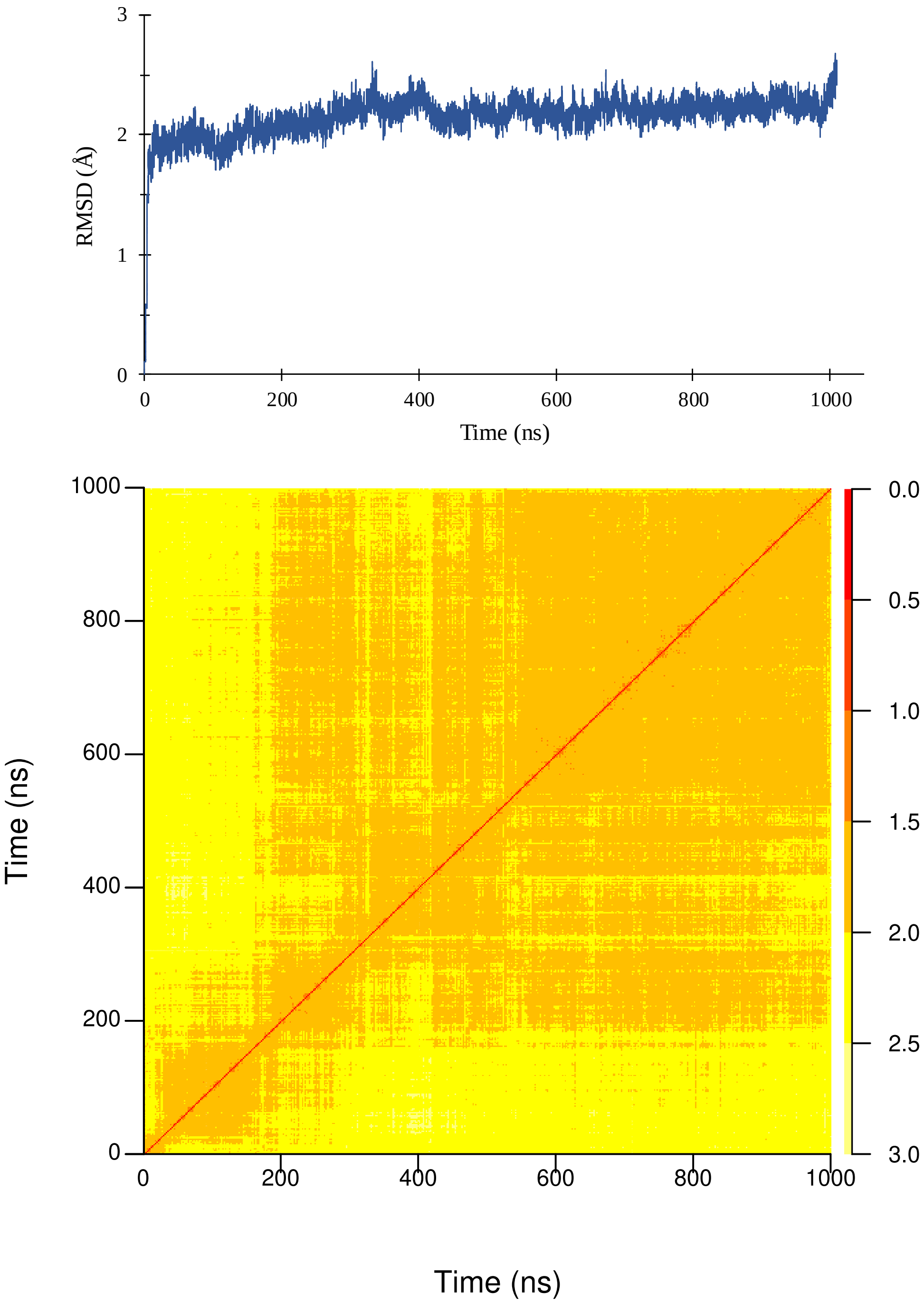

Figure S10. All-atom RMSD plots of EBFP (HID version): 1-D on top and 2-D on the bottom. The 1-D RMS is determined relative to the minimized crystal structure and includes equilibration. The first frame of the 2-D RMS is after 10 ns of equilibration.

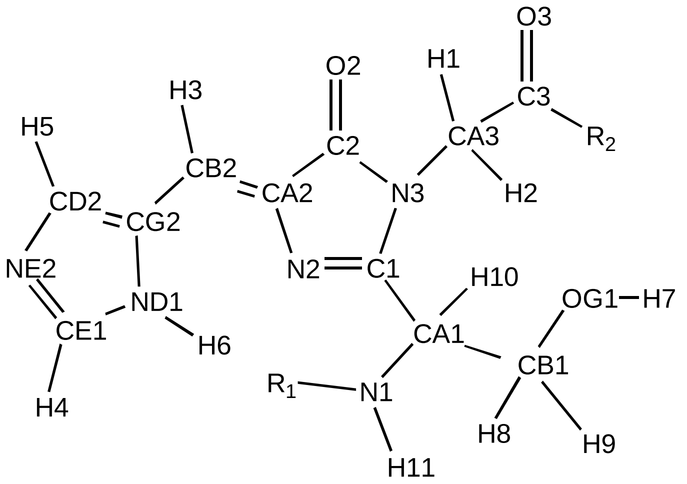

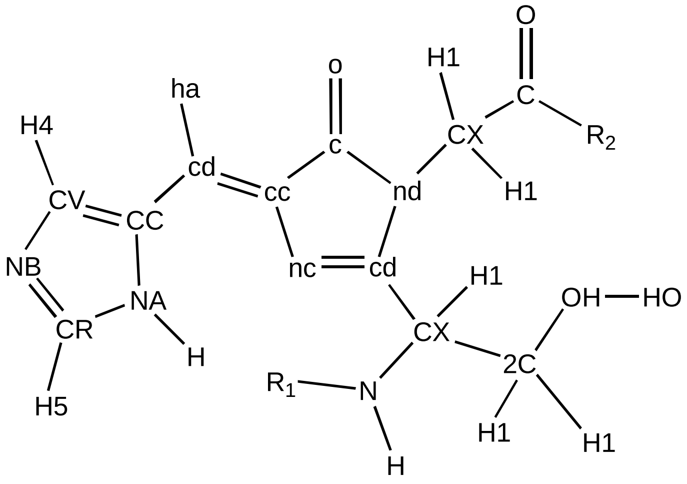

Figure S11. Atom names (top) and atom types (bottom) for the EBFP chromophore with histidine assignment HID.

Table S6. Atom names, types, and charges for the HID version of the EBFP chromophore (PDB residue IICD).

Atom Name	Atom Type	Charge
N1	N	-0.415700
CA1	CX	0.226969
C1	cd	0.127972
N2	nc	-0.422253
CA2	cc	0.192616
C2	c	0.405558
O2	o	-0.533064
N3	nd	-0.086409
CA3	CX	-0.121021
C3	C	0.597300
O3	O	-0.567900
H1	H1	0.110119
H2	H1	0.110119
CB2	cd	-0.312173
H3	ha	0.200381
CG2	CC	0.091864
ND1	NA	-0.328471
CE1	CR	0.281540
H4	H5	0.131618
NE2	NB	-0.579483
CD2	CV	0.139857
H5	H4	0.132586
H6	H	0.301198
CB1	2C	0.105851
OG1	OH	-0.631011
H7	HO	0.380209

H8	H1	0.051526
H9	H1	0.051526
H10	H1	0.086776
H11	H	0.271900

Table S7. RMSDs (in Å) of bond lengths of all bonds (upper) just the imidazolidinone (middle), and just the histidine sidechain (lower) for the HID version of the EBFP chromophore. Three different force-field parameter sets are compared to the crystal structure and to different QM-optimized structures. The smallest RMSD value in each row is bold.

	cc/cd bond parameters	CA/CA bond parameters	ce/cf bond parameters
Crystal structure	0.0451	**0.0420**	0.0540
B3LYP/6-31G(d) SMD 1-pentanol	**0.0163**	0.0325	0.0333
B3LYP/6-31G(d) SMD water	**0.0167**	0.0322	0.0333
B3LYP/6-31G(d) vacuum	**0.0168**	0.0332	0.0340
mp2/cc-pvDZ PCM 1-pentanol	**0.0179**	0.0319	0.0296
mp2/cc-pvDZ PCM water	**0.0178**	0.0315	0.0296
mp2/cc-pvDZ vacuum	**0.0192**	0.0326	0.0309

Imidazolidinone Only

	cc/cd bond parameters	CA/CA bond parameters	ce/cf bond parameters
Crystal structure	0.0531	**0.0511**	0.0553
B3LYP/6-31G(d) SMD 1-pentanol	**0.0158**	0.0380	0.0236
B3LYP/6-31G(d) SMD water	**0.0161**	0.0379	0.0236
B3LYP/6-31G(d) vacuum	**0.0172**	0.0386	0.0251

mp2/cc-pvDZ PCM 1-pentanol	**0.0162**	0.0364	0.0245
mp2/cc-pvDZ PCM water	**0.0162**	0.0361	0.0243
mp2/cc-pvDZ vacuum	**0.0186**	0.0369	0.0266

Histidine Only

	cc/cd bond parameters	CA/CA bond parameters	ce/cf bond parameters
Crystal structure	0.0214	**0.0094**	0.0513
B3LYP/6-31G(d) SMD 1-pentanol	0.0174	**0.0165**	0.0470
B3LYP/6-31G(d) SMD water	0.0177	**0.0156**	0.0470
B3LYP/6-31G(d) vacuum	**0.0159**	0.0183	0.0470
mp2/cc-pvDZ PCM 1-pentanol	0.0208	**0.0201**	0.0379
mp2/cc-pvDZ PCM water	0.0205	**0.0189**	0.0381
mp2/cc-pvDZ vacuum	**0.0204**	0.0216	0.0380

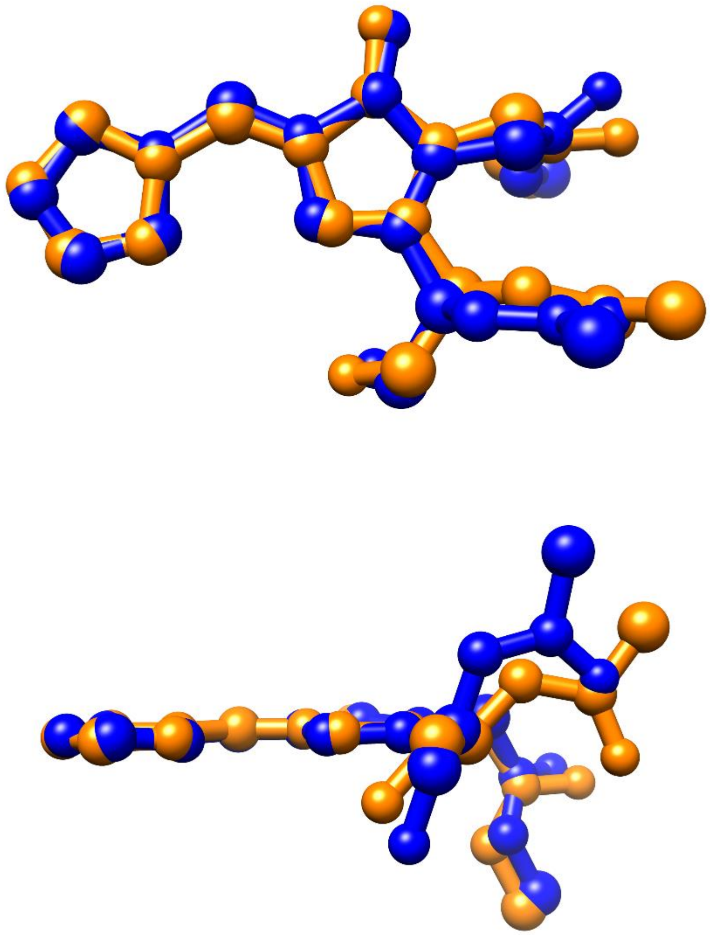

Figure S12. Comparison of PDB and quantum mechanically optimized structures for the HIE form of the EBFP chromophore. The upper and lower images are the same except they are rotated roughly 90 degrees. The PDB structure is in blue and the optimized structure in orange. A ball and stick representation was used where the atoms (balls) are scaled to 25% of the Van der Walls size and the bonds (sticks) are cylinders with 0.2 Å radii.

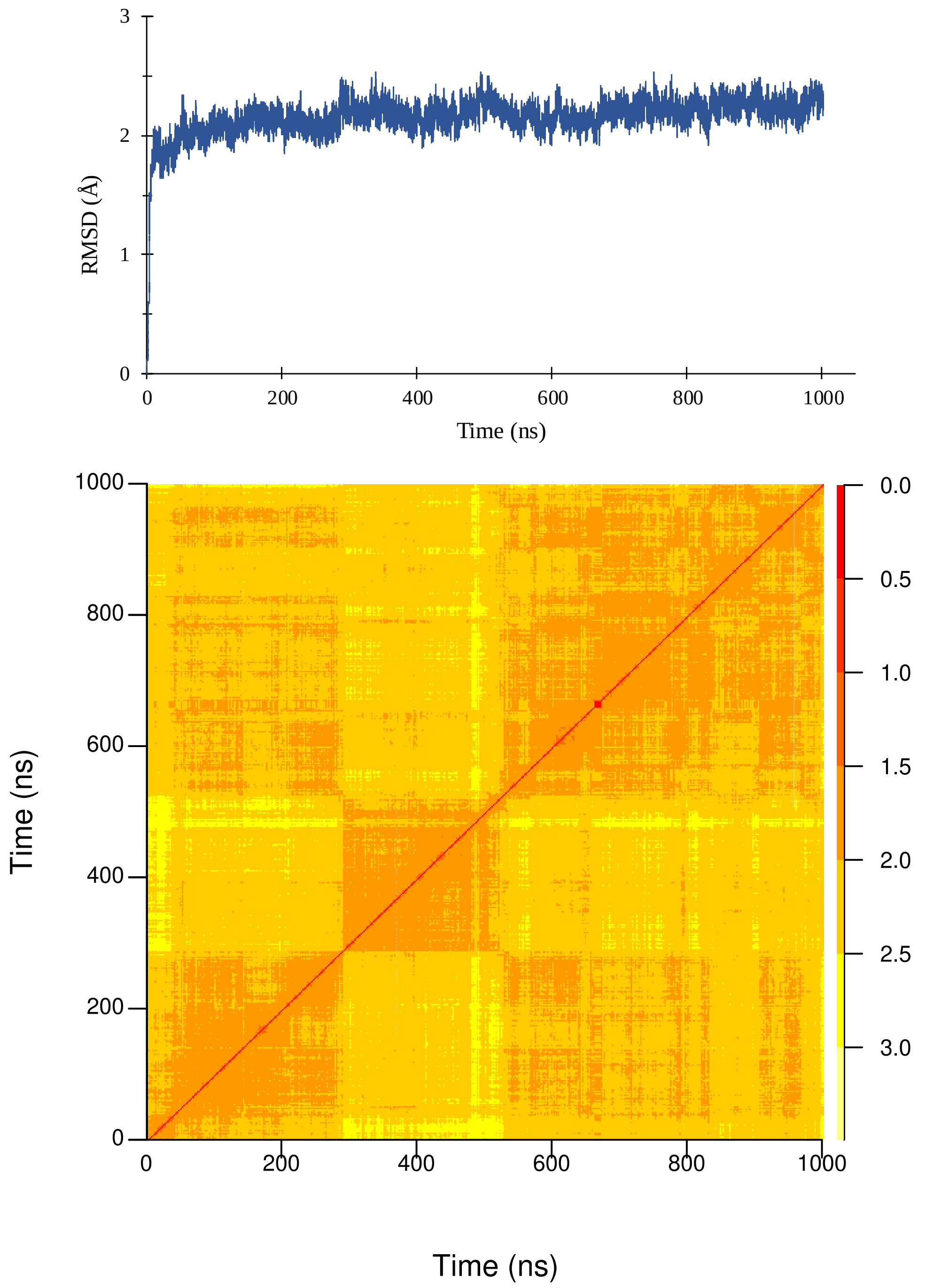

Figure S13. All-atom RMSD plots of EBFP (HIE version): 1-D on top and 2-D on the bottom. The 1-D RMS is determined relative to the minimized crystal structure and includes equilibration. The first frame of the 2-D RMS is after 10 ns of equilibration.

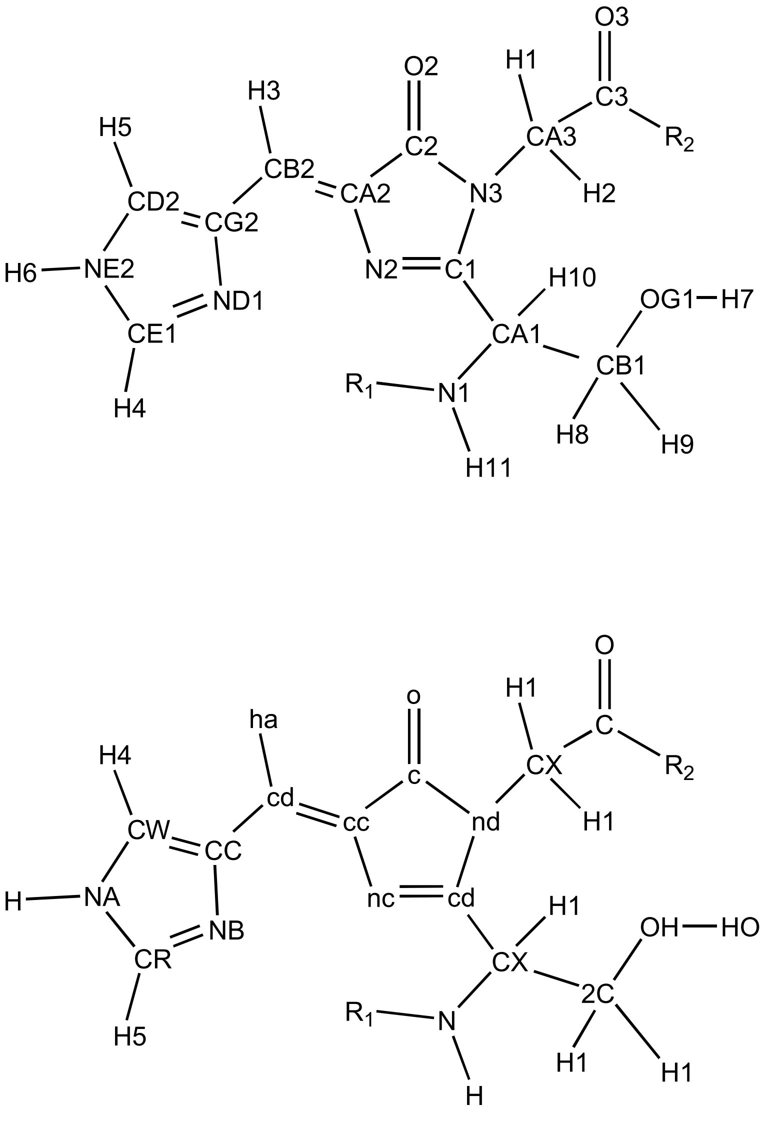

Figure S14. Atom names (top) and atom types (bottom) for the EBFP chromophore with histidine assignment HIE.

Table S8. Atom names, types, and charges for the HIE version of the EBFP chromophore (PDB residue IICE).

Atom Name	Atom Type	Charge
N1	N	-0.415700
CA1	CX	0.227128
C1	cd	0.277651
N2	nc	-0.530113
CA2	cc	0.332553
C2	c	0.404658
O2	o	-0.545050
N3	nd	-0.134212
CA3	CX	-0.199507
C3	C	0.597300
O3	O	-0.567900
H1	H1	0.127943
H2	H1	0.127943
CB2	cd	-0.517360
H3	ha	0.208251
CG2	CC	0.572076
ND1	NB	-0.469021
CE1	CR	0.096352
H4	H5	0.166055
NE2	NA	-0.284396
CD2	CW	-0.347342
H5	H4	0.241065
H6	H	0.357584
CB1	2C	-0.003182
OG1	OH	-0.589069
H7	HO	0.356590

H8	H1	0.077361
H9	H1	0.077361
H10	H1	0.083078
H11	H	0.271900

Table S9. RMSDs (in Å) of bond lengths of all bonds (upper) just the imidazolidinone (middle), and just the histidine sidechain (lower) for the HIE version of the EBFP chromophore. Three different force-field parameter sets are compared to the crystal structure and to different QM-optimized structures. The smallest RMSD value in each row is bold.

	cc/cd bond parameters	CA/CA bond parameters	ce/cf bond parameters
Crystal structure	0.0448	**0.0418**	0.0540
B3LYP/6-31G(d) SMD 1-pentanol	**0.0157**	0.0348	0.0344
B3LYP/6-31G(d) SMD water	**0.0178**	0.0377	0.0342
B3LYP/6-31G(d) vacuum	**0.0172**	0.0377	0.0352
mp2/cc-pvDZ PCM 1-pentanol	**0.0175**	0.0337	0.0300
mp2/cc-pvDZ PCM water	**0.0174**	0.0330	0.0301
mp2/cc-pvDZ vacuum	**0.0188**	0.0362	0.0306

Imidazolidinone Only

	cc/cd bond parameters	CA/CA bond parameters	ce/cf bond parameters
Crystal structure	0.0282	**0.0261**	0.0306
B3LYP/6-31G(d) SMD 1-pentanol	**0.0162**	0.0400	0.0218
B3LYP/6-31G(d) SMD water	**0.0190**	0.0437	0.0214
B3LYP/6-31G(d) vacuum	**0.0191**	0.0436	0.0227

mp2/cc-pvDZ PCM 1-pentanol	**0.0170**	0.0385	0.0229
mp2/cc-pvDZ PCM water	**0.0168**	0.0378	0.0230
mp2/cc-pvDZ vacuum	**0.0197**	0.0417	0.0234

Histidine Only

	cc/cd bond parameters	CA/CA bond parameters	ce/cf bond parameters
Crystal structure	0.002	**0.0001**	0.0132
B3LYP/6-31G(d) SMD 1-pentanol	**0.0145**	0.0210	0.0510
B3LYP/6-31G(d) SMD water	**0.0150**	0.0207	0.0510
B3LYP/6-31G(d) vacuum	**0.0126**	0.0212	0.0518
mp2/cc-pvDZ PCM 1-pentanol	**0.0185**	0.0210	0.0406
mp2/cc-pvDZ PCM water	**0.0186**	0.0205	0.0408
mp2/cc-pvDZ vacuum	**0.0167**	0.0213	0.0415

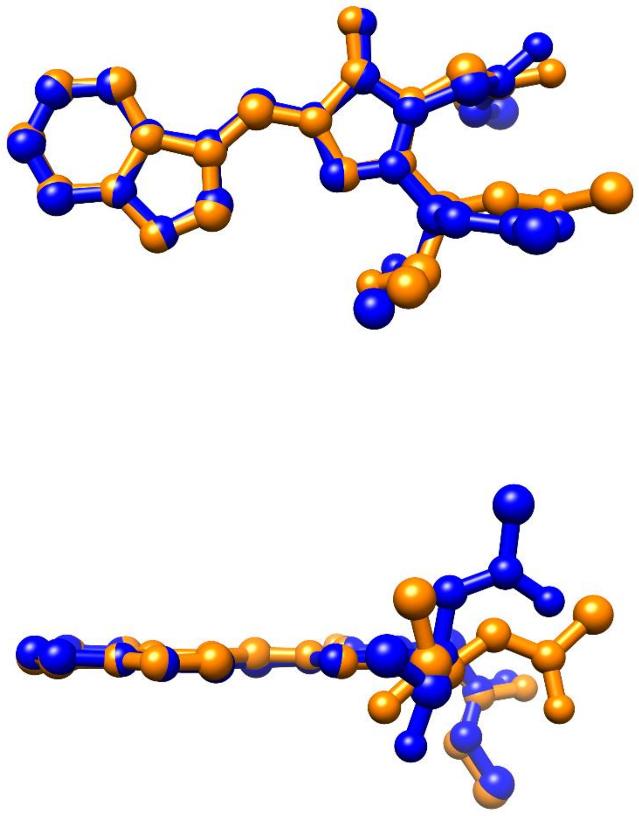

Figure S15. Comparison of PDB and quantum mechanically optimized structures for the ECFP chromophore. The upper and lower images are the same except they are rotated roughly 90 degrees. The PDB structure is in blue and the optimized structure in orange. A ball and stick representation was used where the atoms (balls) are scaled to 25% of the Van der Walls size and the bonds (sticks) are cylinders with 0.2 Å radii.

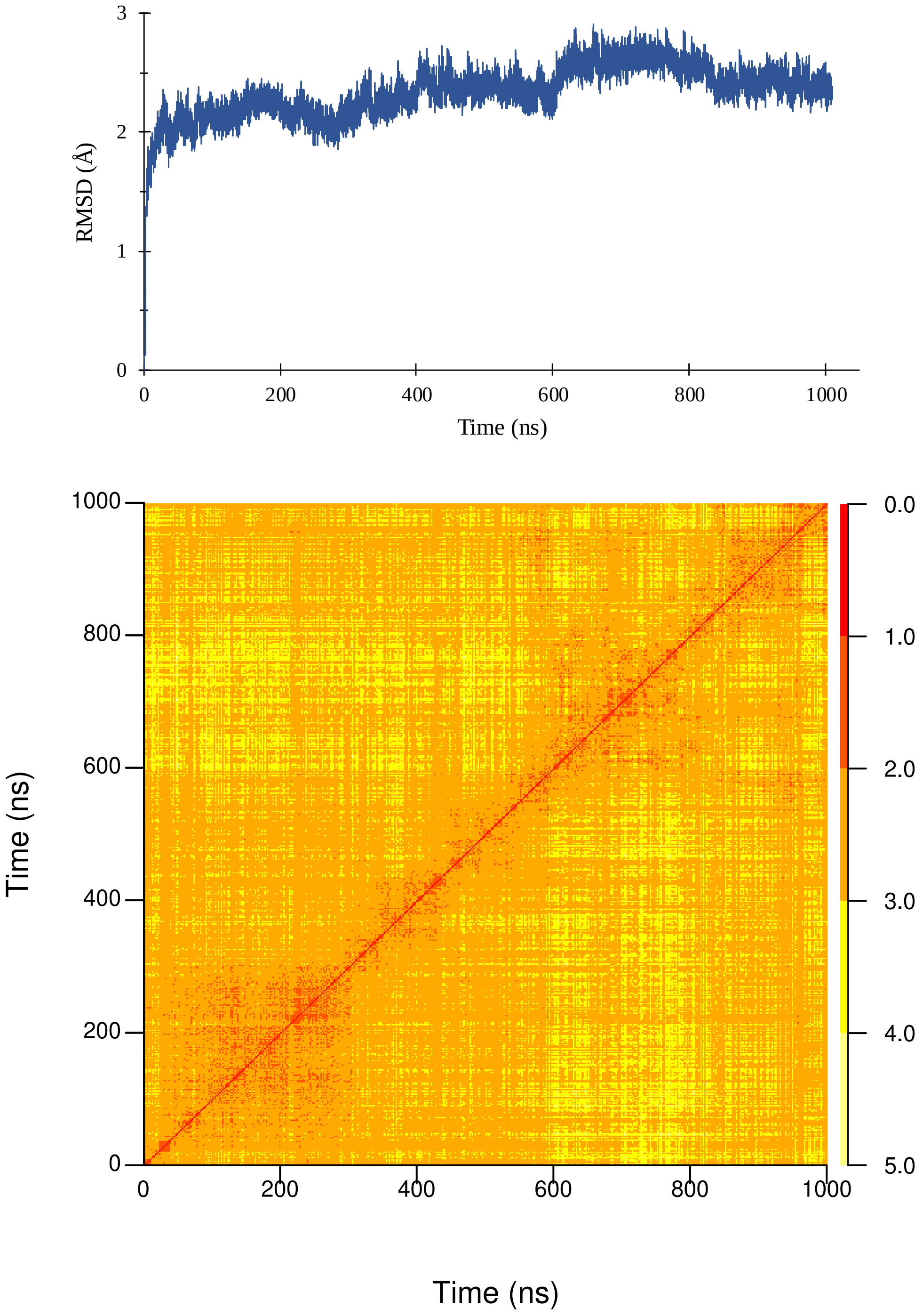

Figure S16. All-atom RMSD plots of ECFP: 1-D on top and 2-D on the bottom. The 1-D RMS is determined relative to the minimized crystal structure and includes equilibration. The first frame of the 2-D RMS is after 10 ns of equilibration.

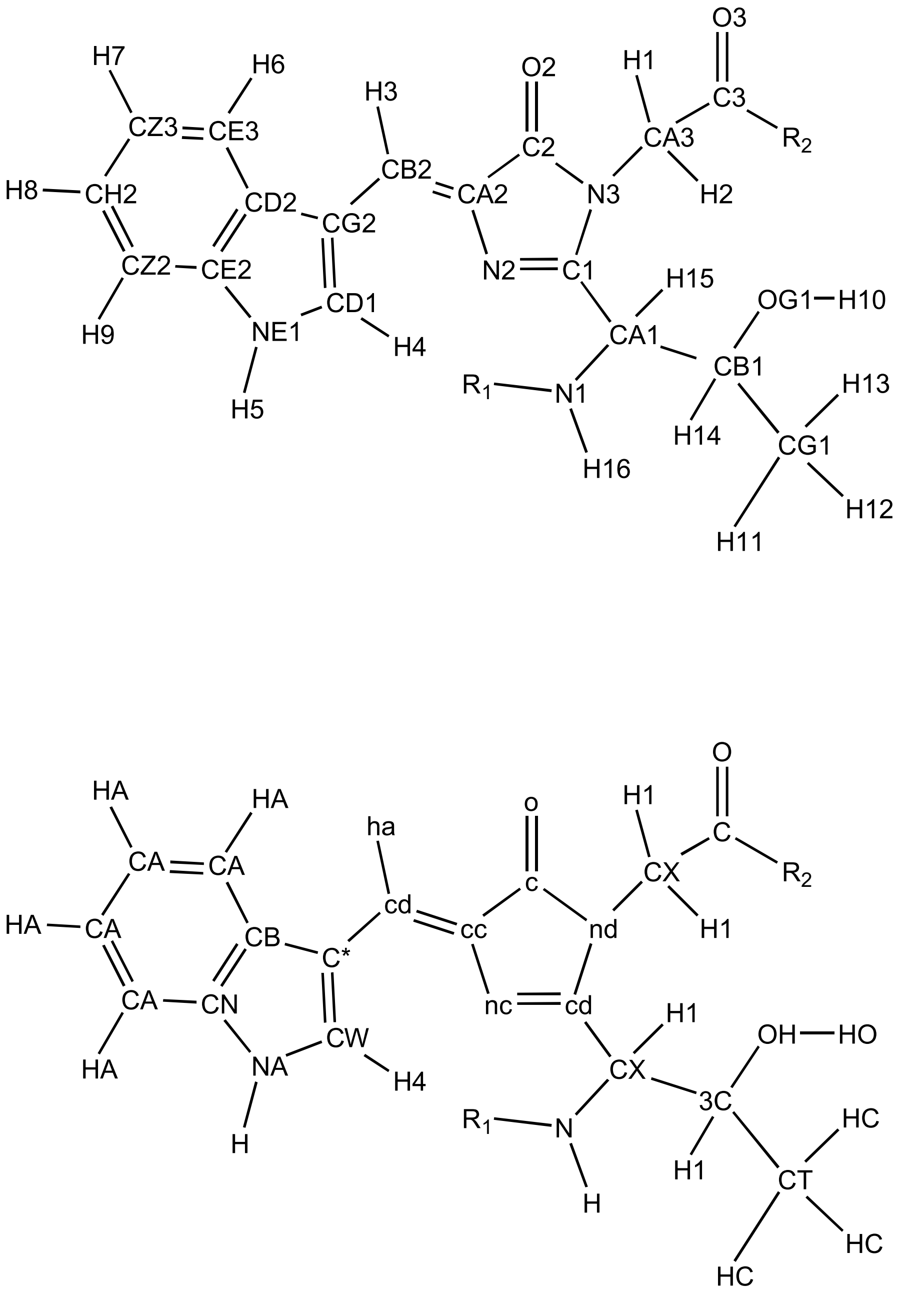

Figure S17. Atom names (top) and atom types (bottom) for the ECFP chromophore.

Table S10. Atom names, types, and charges for the ECFP chromophore (PDB residue CRF).

Atom Name	Atom Type	Charge
N1	N	-0.415700
CA1	CX	0.234489
C1	cd	0.225595
N2	nc	-0.466791
CA2	cc	0.012221
C2	c	0.392369
O2	o	-0.553004
N3	nd	0.012573
CA3	CX	-0.226662
C3	C	0.597300
O3	O	-0.567900
H1	H1	0.121025
H2	H1	0.121025
CB2	cd	-0.087915
H3	ha	0.168079
CG2	C*	-0.062844
CD2	CB	0.030359
CE2	CN	0.294678
NE1	NA	-0.501967
CD1	CW	-0.016836
H4	H4	0.215642
H5	H	0.396580
CZ2	CA	-0.330230
CH2	CA	-0.107971
CZ3	CA	-0.199082
CE3	CA	-0.199959

H6	HA	0.169679
H7	HA	0.156056
H8	HA	0.147378
H9	HA	0.186113
CB1	3C	0.335913
OG1	OH	-0.677925
H10	HO	0.355106
CG1	CT	-0.397952
H11	HC	0.092831
H12	HC	0.092831
H13	HC	0.092831
H14	H1	0.056472
H15	H1	0.033691
H16	H	0.271900

Table S11. RMSDs (in Å) of bond lengths of all bonds (upper) just the imidazolidinone (middle), and just the tryptophan sidechain (lower) for the ECFP chromophore. Three different force-field parameter sets are compared to the crystal structure and to different QM-optimized structures. The smallest RMSD value in each row is bold.

	cc/cd bond parameters	CA/CA bond parameters	ce/cf bond parameters
Crystal structure	**0.0264**	0.0285	0.0432
B3LYP/6-31G(d) SMD 1-pentanol	**0.0195**	0.0290	0.0352
B3LYP/6-31G(d) SMD water	**0.01964**	0.0286	0.0354
B3LYP/6-31G(d) vacuum	**0.0204**	0.0299	0.0354
mp2/cc-pvDZ PCM 1-pentanol	**0.0167**	0.0295	0.0317
mp2/cc-pvDZ PCM water	**0.0165**	0.0291	0.0318
mp2/cc-pvDZ vacuum	**0.0179**	0.0301	0.0322

Imidazolidinone Only

	cc/cd bond parameters	CA/CA bond parameters	ce/cf bond parameters
Crystal structure	0.0381	**0.0375**	0.0233
B3LYP/6-31G(d) SMD 1-pentanol	**0.0165**	0.0371	0.0244
B3LYP/6-31G(d) SMD water	**0.0167**	0.0364	0.0250
B3LYP/6-31G(d) vacuum	**0.0183**	0.0395	0.0249
mp2/cc-pvDZ PCM 1-pentanol	**0.0160**	0.0358	0.0243

mp2/cc-pvDZ PCM water	**0.0159**	0.0351	0.0244
mp2/cc-pvDZ vacuum	**0.0183**	0.0377	0.0253

Tryptophan Only

	cc/cd bond parameters	CA/CA bond parameters	ce/cf bond parameters
Crystal structure	0.0413	**0.0210**	0.0676
B3LYP/6-31G(d) SMD 1-pentanol	0.0221	**0.0175**	0.0433
B3LYP/6-31G(d) SMD water	0.0222	**0.0178**	0.0433
B3LYP/6-31G(d) vacuum	0.0224	**0.0153**	0.0434
mp2/cc-pvDZ PCM 1-pentanol	**0.0172**	0.0213	0.0377
mp2/cc-pvDZ PCM water	**0.0172**	0.0213	0.0378
mp2/cc-pvDZ vacuum	**0.0174**	0.0199	0.0378

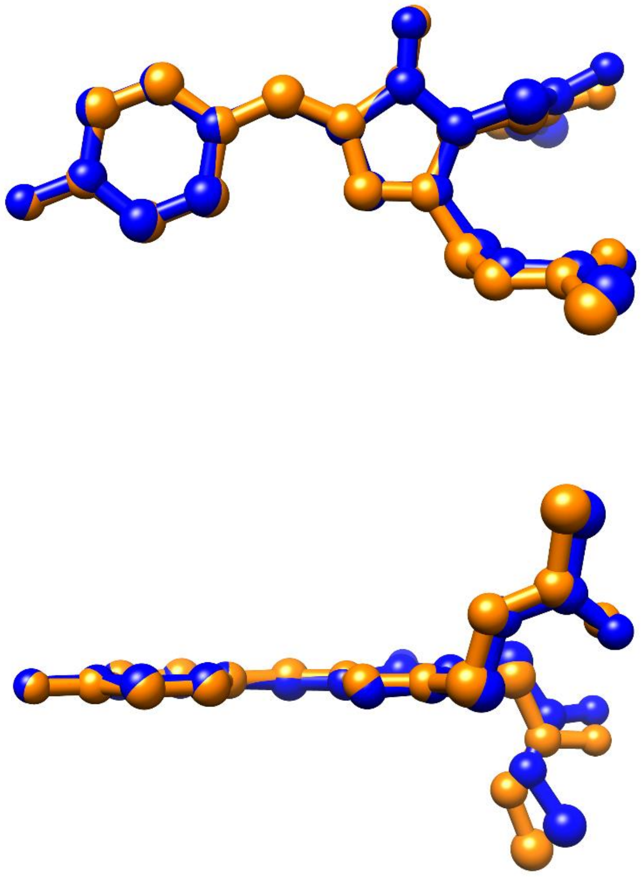

Figure S18. Comparison of PDB and quantum mechanically optimized structures for the EYFP chromophore. The upper and lower images are the same except they are rotated roughly 90 degrees. The PDB structure is in blue and the optimized structure in orange. A ball and stick representation was used where the atoms (balls) are scaled to 25% of the Van der Walls size and the bonds (sticks) are cylinders with 0.2 Å radii.

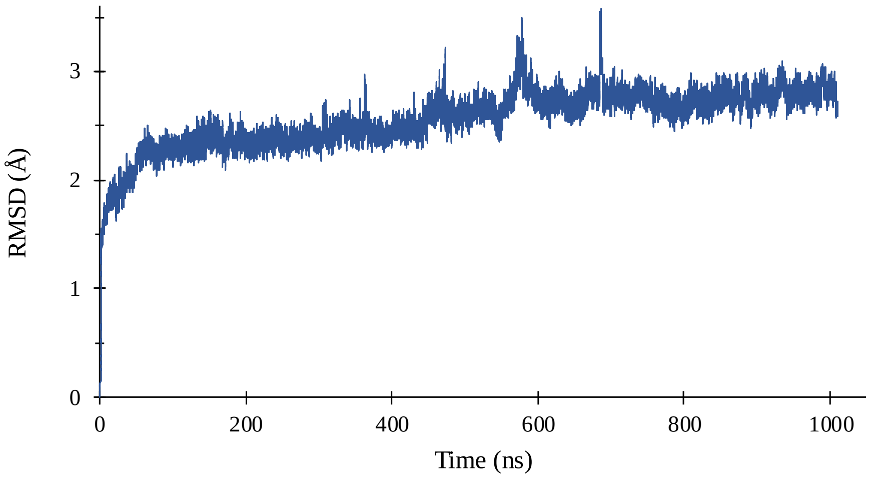

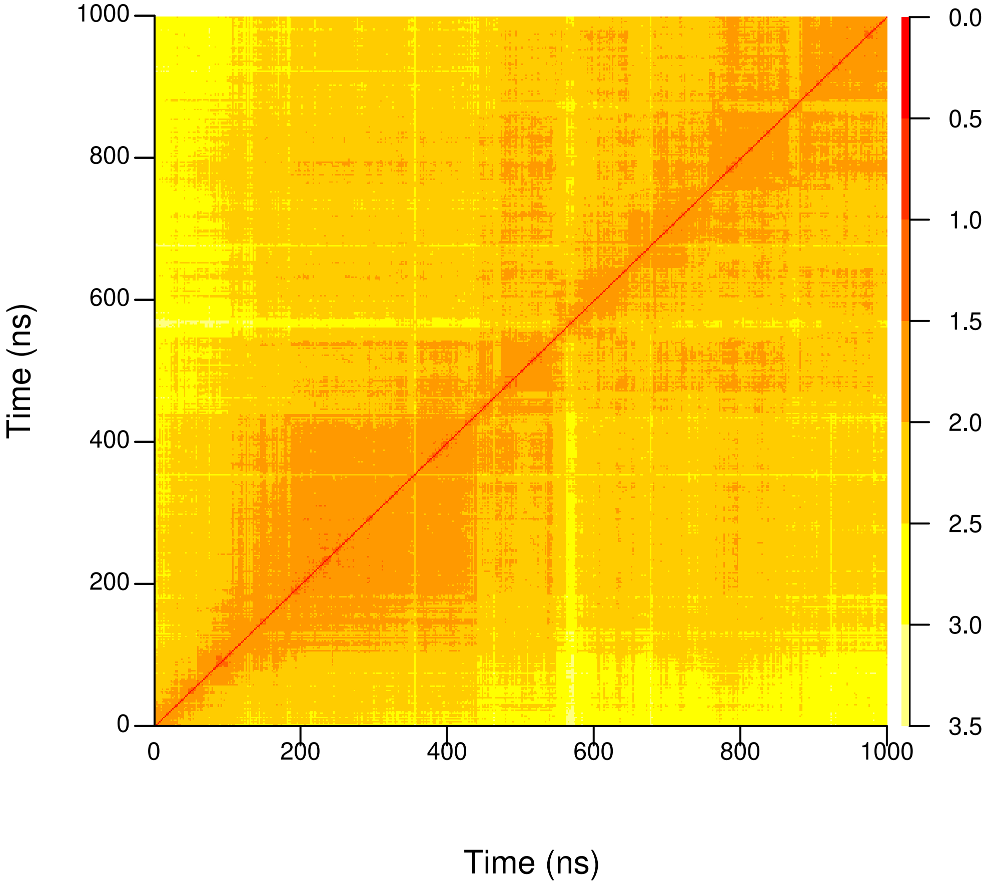

Figure S19. All-atom RMSD plots of EYFP: 1-D on top and 2-D on the bottom. The 1-D RMS is determined relative to the minimized crystal structure and includes equilibration. The first frame of the 2-D RMS is after 10 ns of equilibration.

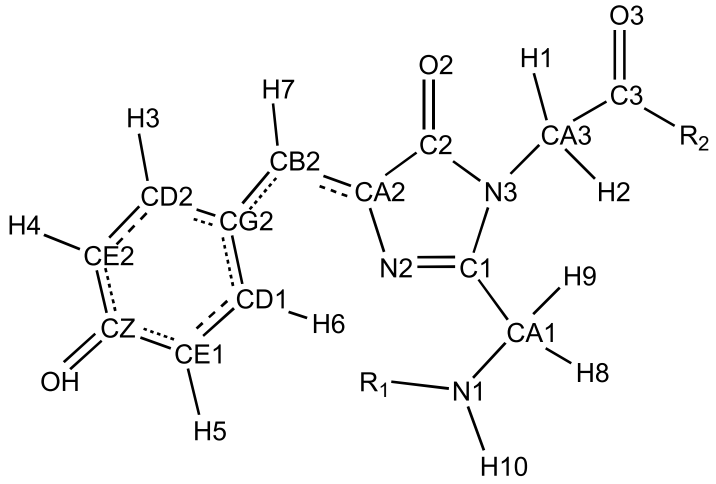

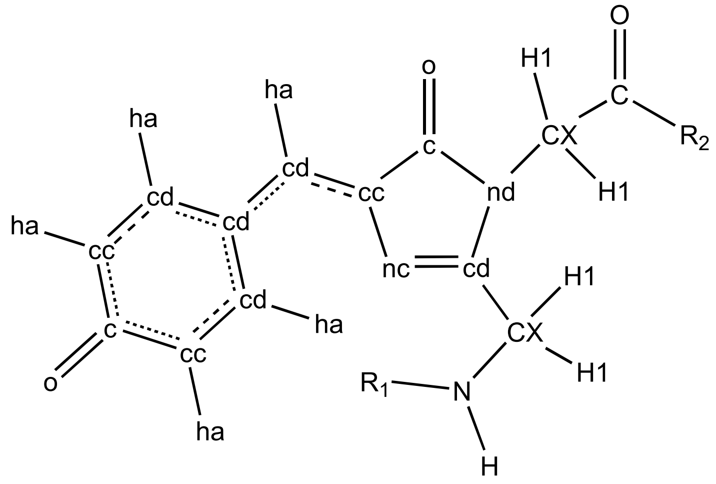

Figure S20. Atom names (top) and atom types (bottom) for the EYFP chromophore.

Table S12. Atom names, types, and charges for the EYFP chromophore (PDB residue CR2).

Atom Name	Atom Type	Charge
N1	N	-0.415700
CA1	CX	0.119283
C1	cd	0.038699
N2	nc	-0.397291
CA2	cc	-0.013775
C2	c	0.350907
O2	o	-0.612651
N3	nd	0.114401
CA3	CX	-0.421980
C3	C	0.597300
O3	O	-0.567900
H1	H1	0.180986
H2	H1	0.180986
CB2	cd	-0.123019
CG2	cd	-0.060785
CD1	cd	0.060679
CE1	cc	-0.551696
CZ	c	0.800552
CE2	cc	-0.495252
CD2	cd	-0.098082
H3	ha	0.138789
H4	ha	0.154946
OH	o	-0.745364
H5	ha	0.168017
H6	ha	0.084510
H7	ha	0.143099
H8	H1	0.049220
H9	H1	0.049220
H10	H	0.271900

Table S13. RMSDs (in Å) of bond lengths of just the imidazolidinone, bridging, and tyrosine-sidechain portions of the chromophore for the EYFP chromophore. Three different force-field parameter sets are compared to the crystal structure and to different QM-optimized structures. The smallest RMSD value in each row is bold.

	cc/cd bond parameters	CA/CA bond parameters	ce/cf bond parameters
Crystal structure	**0.0224**	0.0431	0.0380
B3LYP/6-31G(d) SMD 1-pentanol	**0.0251**	0.0349	0.0314
B3LYP/6-31G(d) SMD water	**0.0236**	0.0347	0.0307
B3LYP/6-31G(d) vacuum	**0.0314**	0.0374	0.0356
mp2/cc-pvDZ PCM 1-pentanol	**0.0258**	0.0337	0.0322
mp2/cc-pvDZ PCM water	**0.0242**	0.0337	0.0314
mp2/cc-pvDZ vacuum	**0.0321**	0.0360	0.0367

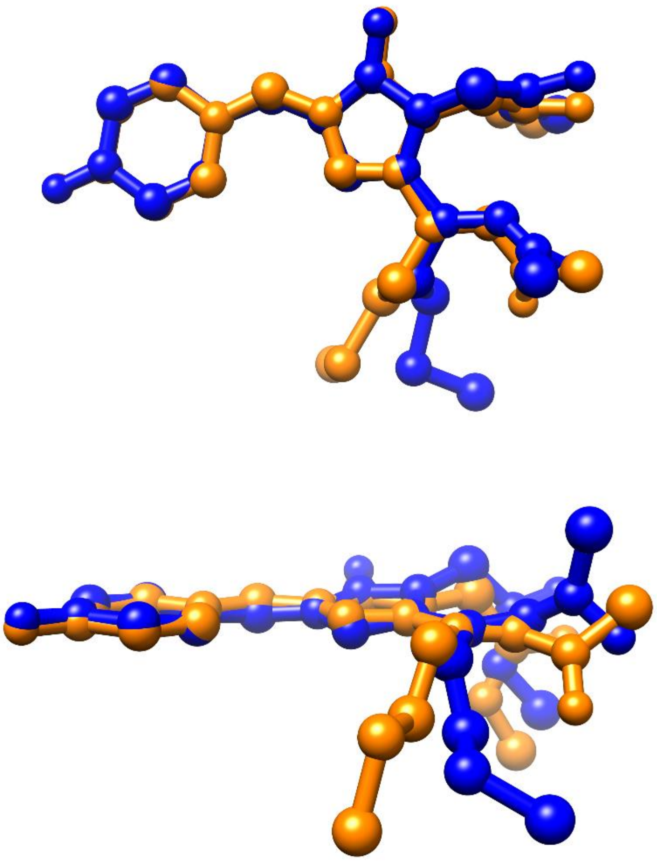

Figure S21. Comparison of PDB and quantum mechanically optimized structures for the mCherry chromophore. The upper and lower images are the same except they are rotated roughly 90 degrees. The PDB structure is in blue and the optimized structure in orange. A ball and stick representation was used where the atoms (balls) are scaled to 25% of the Van der Walls size and the bonds (sticks) are cylinders with 0.2 Å radii.

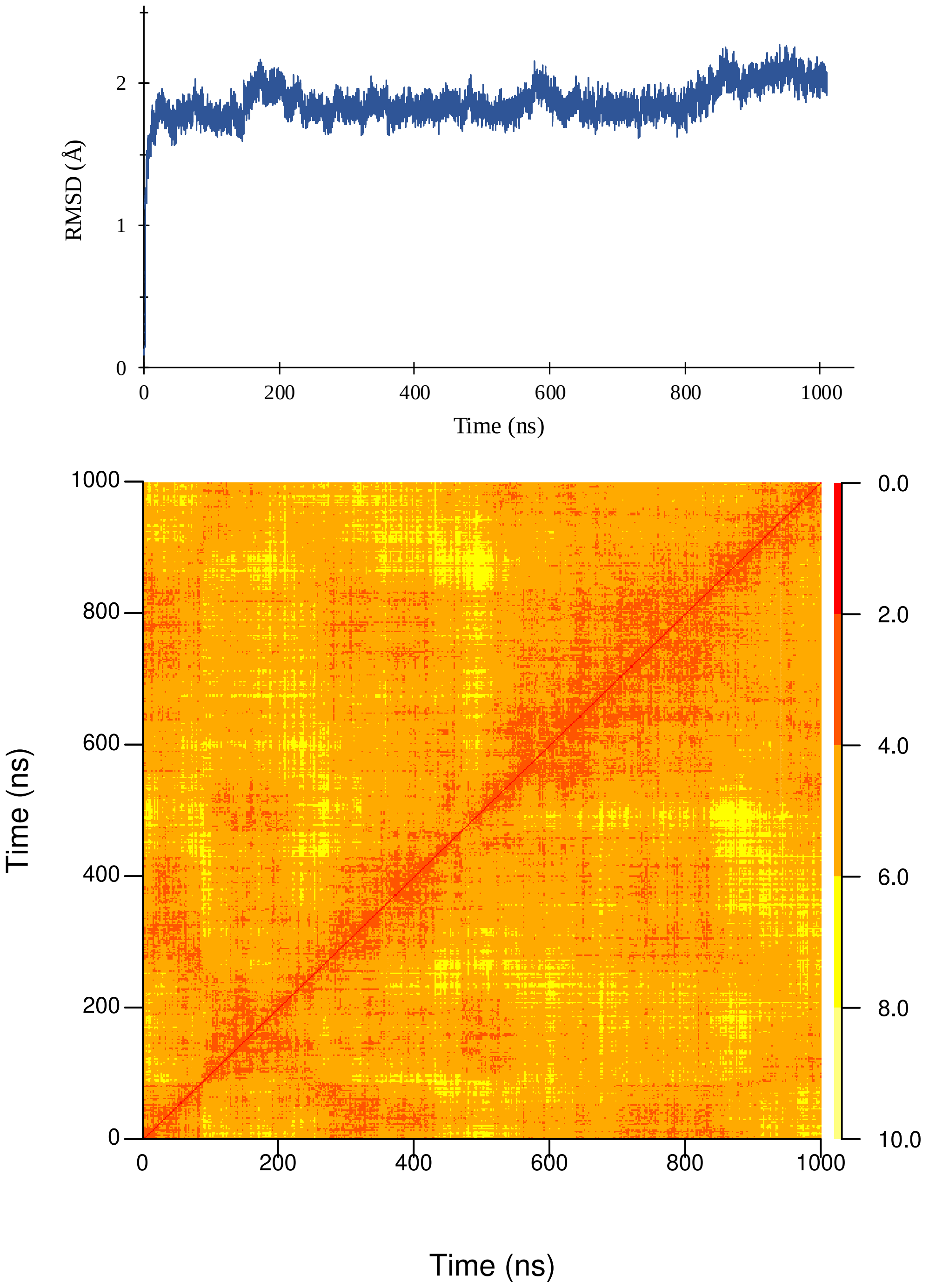

Figure S22. All-atom RMSD plots of mCherry: 1-D on top and 2-D on the bottom. The 1-D RMS is determined relative to the minimized crystal structure and includes equilibration. The first frame of the 2-D RMS is after 10 ns of equilibration.

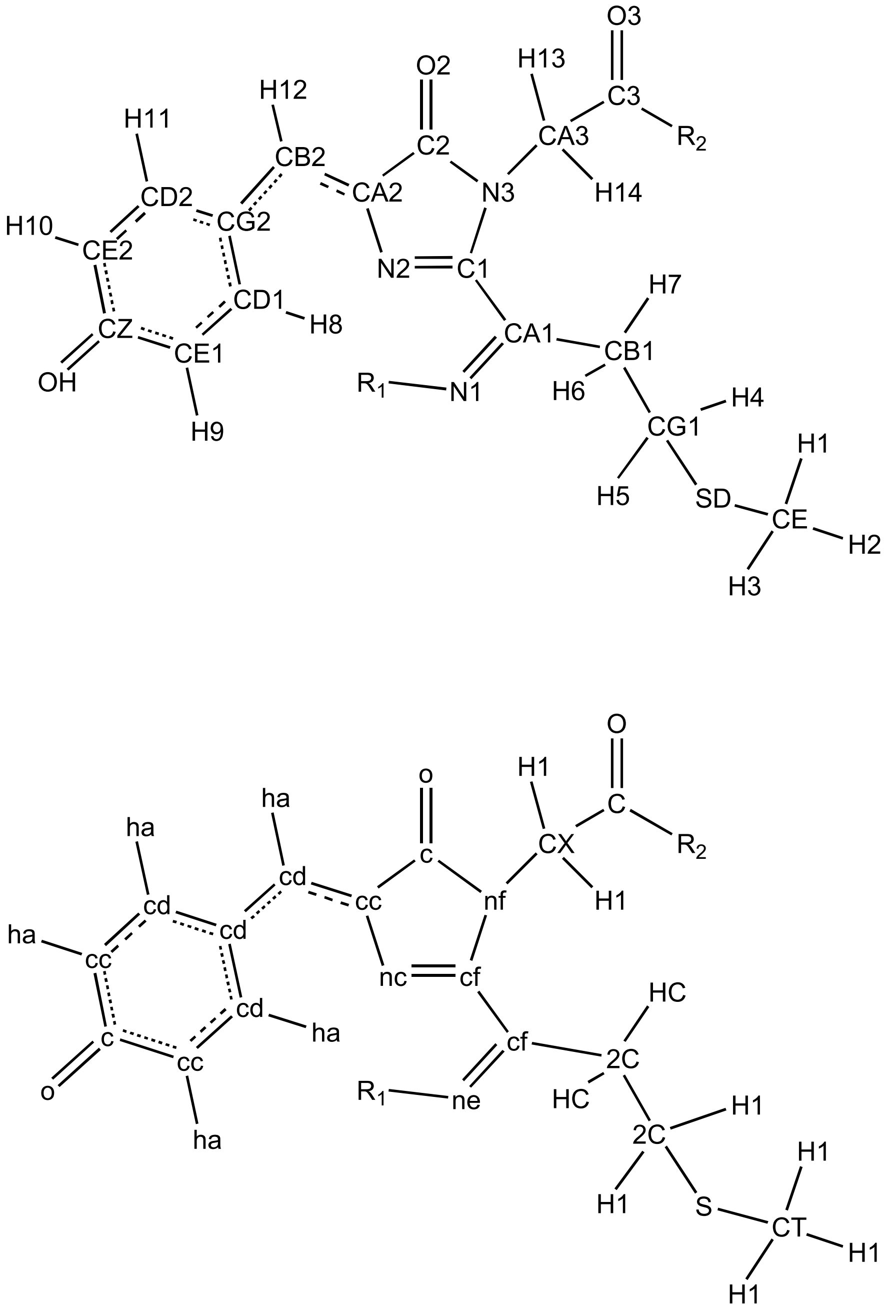

Figure S23. Atom names (top) and atom types (bottom) for the mCherry chromophore.

Table S14. Atom names, types, and charges for the mCherry chromophore (PDB residue CH6).

Atom Name	Atom Type	Charge
N1	ne	-0.283649
CA1	cf	0.147450
CB1	2C	-0.094731
CG1	2C	-0.288860
SD	S	-0.236004
CE	CT	-0.264339
H1	H1	0.123939
H2	H1	0.123939
H3	H1	0.123939
H4	H1	0.177239
H5	H1	0.177239
H6	HC	0.111880
H7	HC	0.111880
C1	cf	0.028005
N2	nc	-0.326404
CA2	cc	-0.044694
CB2	cd	-0.136608
CG2	cd	-0.014736
CD2	cd	-0.105062
CE2	cc	-0.480682
CZ	c	0.806381
CE1	cc	-0.491765
CD1	cd	0.008631
H8	ha	0.115035
H9	ha	0.155233
OH	o	-0.716610

H10	ha	0.159963
H11	ha	0.140719
H12	ha	0.159216
C2	c	0.419145
O2	o	-0.655758
N3	nf	0.140441
CA3	CX	-0.471265
C3	C	0.597300
O3	O	-0.567900
H13	H1	0.175747
H14	H1	0.175747

Table S15. RMSDs (in Å) of bond lengths of just the imidazolidinone, bridging, and tyrosine-sidechain portions of the chromophore for mCherry. Three different force-field parameter sets are compared to the crystal structure and to different QM-optimized structures. The smallest RMSD value in each row is bold.

	cc/cd bond parameters	CA/CA bond parameters	ce/cf bond parameters
Crystal structure	**0.0379**	0.0389	0.0380
B3LYP/6-31G(d) SMD 1-pentanol	**0.0276**	0.0346	0.0334
B3LYP/6-31G(d) SMD water	**0.0262**	0.0365	0.0320
B3LYP/6-31G(d) vacuum	**0.0326**	0.0328	0.0380
mp2/cc-pvDZ PCM 1-pentanol	**0.0289**	0.0326	0.0350
mp2/cc-pvDZ PCM water	**0.0271**	0.0344	0.0335
mp2/cc-pvDZ vacuum	**0.0342**	0.0346	0.0409
